# Macrophage-Tumor Cell Fusions from Peripheral Blood of Melanoma Patients

**DOI:** 10.1371/journal.pone.0134320

**Published:** 2015-08-12

**Authors:** Gary A. Clawson, Gail L. Matters, Ping Xin, Yuka Imamura-Kawasawa, Zhen Du, Diane M. Thiboutot, Klaus F. Helm, Rogerio I. Neves, Thomas Abraham

**Affiliations:** 1 Department of Pathology and Gittlen Cancer Research Laboratories, Hershey Medical Center, Pennsylvania State University, Hershey, Pennsylvania, United States of America; 2 Department of Biochemistry & Molecular Biology, Hershey Medical Center, Pennsylvania State University, Hershey, Pennsylvania, United States of America; 3 Department of Pharmacology and the Institute for Personalized Medicine, Hershey Medical Center, Pennsylvania State University, Hershey, Pennsylvania, United States of America; 4 Department of Dermatology, Division of Health Science Research, Hershey Medical Center, Pennsylvania State University, Hershey, Pennsylvania, United States of America; 5 Department of Dermatopathology, Hershey Medical Center, Pennsylvania State University, Hershey, Pennsylvania, United States of America; 6 Department of Surgery and the Melanoma Center, Hershey Medical Center, Pennsylvania State University, Hershey, Pennsylvania, United States of America; 7 Department of Neural and Behavioral Science and the Microscopy Imaging Facility, Hershey Medical Center, Pennsylvania State University, Hershey, Pennsylvania, United States of America; Ospedale Pediatrico Bambino Gesu', ITALY

## Abstract

**Background:**

While the morbidity and mortality from cancer are largely attributable to its metastatic dissemination, the integral features of the cascade are not well understood. The widely accepted hypothesis is that the primary tumor microenvironment induces the epithelial-to-mesenchymal transition in cancer cells, facilitating their escape into the bloodstream, possibly accompanied by cancer stem cells. An alternative theory for metastasis involves fusion of macrophages with tumor cells (MTFs). Here we culture and characterize apparent MTFs from blood of melanoma patients.

**Methods:**

We isolated enriched CTC populations from peripheral blood samples from melanoma patients, and cultured them. We interrogated these cultured cells for characteristic BRAF mutations, and used confocal microscopy for immunophenotyping, motility, DNA content and chromatin texture analyses, and then conducted xenograft studies using nude mice.

**Findings:**

Morphologically, the cultured MTFs were generally large with many pseudopod extensions and lamellipodia. Ultrastructurally, the cultured MTFs appeared to be macrophages. They were rich in mitochondria and lysosomes, as well as apparent melanosomes. The cultured MTF populations were all heterogeneous with regard to DNA content, containing aneuploid and/or high-ploidy cells, and they typically showed large sheets (and/or clumps) of cytoplasmic chromatin. This cytoplasmic DNA was found within heterogeneously-sized autophagic vacuoles, which prominently contained chromatin and micronuclei. Cultured MTFs uniformly expressed pan-macrophage markers (CD14, CD68) and macrophage markers indicative of M2 polarization (CD163, CD204, CD206). They also expressed melanocyte-specific markers (ALCAM, MLANA), epithelial biomarkers (KRT, EpCAM), as well as the pro-carcinogenic cytokine MIF along with functionally related stem cell markers (CXCR4, CD44). MTF cultures from individual patients (5 of 8) contained melanoma-specific BRAF activating mutations. Chromatin texture analysis of deconvoluted images showed condensed DNA (DAPI-intense) regions similar to focal regions described in stem cell fusions. MTFs were readily apparent in vivo in all human melanomas examined, often exhibiting even higher DNA content than the cultured MTFs. When cultured MTFs were transplanted subcutaneously in nude mice, they disseminated and produced metastatic lesions at distant sites.

**Conclusions and Hypothesis:**

Apparent MTFs are present in peripheral blood of patients with cutaneous melanomas, and they possess the ability to form metastatic lesions when transplanted into mice. We hypothesize that these MTFs arise at the periphery of primary tumors in vivo, that they readily enter the bloodstream and invade distant tissues, secreting cytokines (such as MIF) to prepare “niches” for colonization by metastasis initiating cells.

## Introduction

While the morbidity and mortality from cancer are largely attributable to its metastatic dissemination, the integral components/features of the metastatic cascade are not well understood. The most widely accepted hypothesis underlying metastasis is that the primary tumor microenvironment (TME) induces an epithelial-to-mesenchymal transition (EMT) in a subset of epithelial cancer cells, that confers increased motility and invasiveness and facilitates their escape into the bloodstream[1]. A number of studies lend support to this conjecture, for example studies that document EMT-related changes (and loss of EpCAM expression) in circulating tumor cells (CTCs) [2–6]. In spite of recognized shortcomings [7, 8] considerable evidence has accumulated showing that numbers of EpCAM+ CTCs in peripheral blood has prognostic significance for patients [9–11]. However, the picture remains incomplete in a number of areas. One vexing question is which CTCs are the capable of initiating metastatic lesions (so called metastasis initiating cells, MICs) and another is how MICs find suitable landing places [1]. With regard to the former, a corollary idea is that the EMT-altered cancer cells at the periphery of a primary tumor facilitate liberation of cancer stem cells with them [1, 12, 13], which would represent the MICs. Thus, the global level of the CTC population would stochastically represent a much smaller subset of MICs, which presumably arise from a competitive hierarchy of subpopulations of genetically diverse cancer stem cells [14]. However, this story does not address the latter question, how MICs find suitable “niches” which allow them to establish metastases and proliferate [15]. Certainly exosomes could play a part in preparing adjacent tissues (for example, sentinel lymph nodes; [16]), but significant concentrations of exosomes at distant sites are more difficult to envision.

An alternative theory for metastasis [17, 18] involves fusion of macrophages with tumor cells (macrophage-tumor cell fusions, MTFs). With some sort of recombination/reprogramming [19] of genetic material, perhaps analogous to that being studied in stem cell fusions [20–22] of genetic material, this could produce neoplastic cells which have acquired “professional grade” invasive properties characteristic of macrophages. Indeed, there are suggestions that the EMT might better be described as an epithelial-myeloid transition [23]. There is considerable support for this notion from animal models, and some recent support from reports of human cancers, but how frequently this occurs is unknown and the basic premise seems to be at odds with the EMT/stem cell hypothesis [15]. An intriguing synthesis of these ideas is that MTFs could facilitate development of TMEs at distant sites, potentially addressing the problem of how MICs find suitable niches for colonization.

Here, we have isolated and cultured MTFs from peripheral blood from several patients with cutaneous melanomas, and describe their various properties, including their ability to disseminate and form metastatic lesions. We hypothesize that these MTFs play an early, integral part in the metastatic cascade. MTFs formed at the periphery of primary tumors could readily enter the bloodstream (or lymphatics) and invade distant tissues, subsequently releasing cytokines, particularly MIF (and potentially exosomes) which prepare these distant sites for colonization by MICs. The MTFs could themselves represent the metastasis initiating cells (MICs) [18, 24], recognizing the potential self-renewal capacities of macrophages [25]. Alternatively, successful MICs could also be disseminating cancer stem cells which evolve within heterogeneous tumors [14], a scenario in which the MTFs would function as a stromal component to produce a TME suitable for metastatic growth.

## Materials and Methods

### Isolation and Culturing of MTFs

This Human Subjects Research was approved by the Pennsylvania State University/Hershey Medical Center IRB, and informed written consent was obtained from all participants.

Peripheral blood was obtained from patients with cutaneous melanomas (or healthy volunteers). An initial CTC enrichment was performed using Oncoquick porous membrane gradients as previously described [26], or using Ficoll-Paque PLUS gradients (GE Healthcare). The CTC-enriched fractions were rinsed in PBS, and then plated onto standard culture dishes and cultured in RPMI 1640 + 10% bovine serum. After 24 hours, plates were carefully rinsed to remove non-adherent cells, and new medium was added and cultures were continued for various periods of time, with the medium changed 2X per week. Many of the cells appeared large and “epithelioid” at 24 h, and retained the same basic morphology throughout culturing; this was also noted in a previous publication which also examined CTCs captured on filters, where “typical” CTCs were also observed [26]. After our initial experiences, the culture protocol was changed to use 25 cm dishes (instead of 75 cm), which seemed to allow better growth with no change in the characteristics of the cultures, and we were able to harvest greater numbers of cells (5x10^5^ cells vs. initial cultures which had yielded ≤ 10^3^−10^4^ cells).

### Subcutaneous implantation of cultured human melanoma MTFs into athymic mice

4 week old athymic nude mice (nu+/nu+)were obtained from the NCI stocks at Charles River Laboratories. After initial acclimation period (7 days), mice were anesthetized with 129 mg/kg Ketamine and 4 mg/kg Xylazine. Once properly anesthetized, mice (2) were injected with 5 x 10^5^ cultured human melanoma MTFs in a volume of 100 μl into a hind flank. Experiments used cultured MTFs from 2 separate melanoma patient samples, which had been grown in culture for ~4 weeks. Mice were monitored for any outward sign of tumor formation; after 47 days, mice were anesthetized and euthanized. A necropsy was performed, and tissues were harvested and fixed in 10% neutral buffered formalin. After 24 hours, tissues were transferred to 70% ethanol (v/v) and embedded in paraffin blocks, sectioned, and stained using immunohistochemistry as described below.

### BRAF Mutational Analyses

Given the high prevalence (~60%) of BRAF mutations in cutaneous melanomas [27], CTC-enriched fractions were analyzed for mutations in the kinase domain of BRAF. Enriched CTC populations from 11 patients were examined for specific activating BRAF V600 mutations, using specific primers. We also analyzed cultured MTF populations derived from 8 patients.

Primers used were: Claw3461 BRAF Normal‐For = 5’‐ACAGGGCATGGATTACTTACA (for all reactions); Claw3462 BRAF Normal‐Rev = 5’‐GGACCCACTCCATCGAGATTTCA; Claw3463 V600E‐R = 5’‐GGACCCACTCCATCGAGATTTCT; Claw3464V600K‐R = 5’‐GGACCCACTCCATCGAGATTTCTT; Claw3465 V600R‐R = 5’‐GGACCCACTCCATCGAGATTTCCT; Claw3466 V600E2‐R = 5’‐GGACCCACTCCATCGAGATTTTT; Claw3467 V600D‐R = 5’‐GGACCCACTCCATCGAGATTTAT; Claw3468 V600E‐F2 = 5’‐GGTGATTTTGGTCTAGCTACAGA; and Claw3469V600E‐R2 = 5’‐TGCATTCTGATGACTTCTGGT. After primer-specific amplifications, products were verified by size, and where necessary by cloning/sequencing.

### Immunofluorescent Staining of MTF Cultures

Immunochemical staining was performed with antibodies against a variety of different markers (see S1 Table). Antibodies specific for human MLANA, CD204, and CD206 were used to unambiguously identify human cells in mouse tissues.

Generally cells were stained for various combinations of 2 markers, as well as with DAPI, and examined by confocal microscopy. The human pancreatic ductal epithelial cell line HPDE (ATCC, CRL-4023) was used as a normal diploid control. Human melanoma cell lines SK-Mel-24 (ATCC, HTB-71, metastatic), SK-Mel-28 (CTRL HTB-72, primary), SK-Mel-31 (CTRL, HTB-73, primary), and the human pancreatic ductal adenocarcinoma cell line Panc-1 (ATCC, CRL-1469) cells were also examined in parallel.

Cells were grown overnight in 8-well coated chamber slides (Lab-Tek II CC^2^) and immunostaining was performed as previously described [26]. Blocking was performed in PBS + 1:50 dilution of serum (from the species the secondary antibody was produced in) for 1 h at RT. 200 μl/chamber well of primary antibody solutions (1:200 dilution) was used. The balance of the staining protocol was performed in the dark, or with slides protected from light. 200 μl of secondary antibody (1:500 dilution in blocking solution) was used. Controls routinely included no primary antibody. To counterstain nuclei, 200 μl of DAPI solution (1:30,000 in PBS) was added to each well, and incubated for 5 min at RT in the dark. Cells were again washed 3X for 3 min each in PBST, and then given a final rinse for 10 min at RT with PBST, and the final wash was removed by inversion wicking. Coverslips were mounted using 3 drops of ProLong Gold Antifade mounting medium (Invitrogen), pre-warmed to RT, and slides were stored in the dark at 4°C. The mounting medium minimizes the refractive index mismatch of the lens immersion liquid (Cargile oil, refractive index ~ 1.52).

### Immunohistochemical Staining

Formalin-fixed paraffin-embedded (FFPE) tissue specimens from 6 primary melanomas, and 2 metastatic melanomas, were obtained under an IRB-approved protocol (without identifiers), and they were also stained for the macrophage, melanocyte, and epithelial markers (and DAPI) described above, and examined by standard light microscopy and/or confocal microscopy. Where appropriate, secondary antibodies and reagents for immunoperoxidase staining were purchased from Vector Laboratories. They were: ImmPress Anti-Rabbi Ig (peroxidase) (MP-7401) and ImmPress Anti-Mouse Ig (peroxidase) (MP-7402). Peroxidase substrate kits used were ImmPACT DAB Substrate (SK-4104)

### Nuclear Staining for Ploidy Analysis

As described above, nuclei of the cultured MTFs from melanoma patients were fluorescently labeled with DAPI (4′,6-diamidino-2-phenylindole, from Invitrogen) and/or with TO-PRO-3 dye; although the latter dye has been reported to be a useful DNA stain [28], in our hands it exhibited even greater photobleaching than with DAPI, in spite of the ability to use pulsed excitation. For tissue sections, control diploid nuclei were imaged from normal, benign regions of the same slides.

### 3D Confocal Microscopy and Image Acquisition

Confocal images of fluorescently labeled cells were acquired with a Leica AOBS SP8 laser scanning confocal microscope (Leica, Heidelberg, Germany) using a high resolution Leica 40X/1.3 Plan-Apochromat oil immersion objective. The laser lines used for excitation were continuous wave 405 (for Dapi), 80 MHz pulsed 499 nm (for Alexa 488), 80 MHz pulsed 591 (for Alexa 594) and 80 MHz pulsed 645 nm (for TO-PRO-3). These laser lines were produced by UV diode, 80 MHz white light laser (Leica AOBS SP8 module) respectively and the respective emission signals were collected sequentially using AOBS tunable filters as follow; 410–480 nm for DAPI (this exclude possible RNA bound DAPI emission which occurs above 500 nm), 504–571 nm for Alexa 488, 597–751 nm for Alexa 594 and 650–790 nm for TO-PRO-3. All images, spectral data and DNA ploidy measurement data were generated using the highly sensitive HyD detectors (with time gated option) in descanned mode and the photon counting mode was particularly used for collecting signals from DAPI and TO-PRO-3 for DNA ploidy measurements. The backscattered emission signals from the sample were delivered through the AOBS tunable filter (to remove irradiated laser), the detection pinhole set to 1 Airy unit (to obtain optimal lateral and axial resolutions), spectral dispersion prism, and finally to the HyD detectors. The width of the slits in front of each HyD could be software adjusted so that each HyD could detect spectral regions spanning from a 10-nm bandwidth up to the overall spectral capacity of the system (400–800 nm). Using this unique option, spectral scanning was performed on all the dyes to confirm signal specificity.

### Deconvolution

*For 3D image data set acquisition*, the excitation beam was first focused at the maximum signal intensity focal position within the sample and the appropriate HyD gain level was then selected to obtain the pixel intensities within range of 0–255 (8-bit images) using a color gradient function. Later on, the beginning and end of the 3D stack (i.e. the top and the bottom optical sections) were set based on the signal level degradation. Series of 2D Images for a selected 3D stack volume were then acquired with 1024X1024 pixels and were line averaged 3–4 times depending on the noise level. The 3D stack images with optical section thickness (z-axis) of approximately 0.3 m were captured from cell volumes. For each cell volume reported here, z-section images were compiled and finally the 3-dimensional image restoration was performed using Imaris software (Bitplane).

### DNA Ploidy Quantification

The DNA ploidy measurement from 3D rendered confocal image dataset is a well-established procedure [29]. Specifically, the computation of DNA voxel intensities was performed on the 3D image data sets recorded from several areas of cell samples using IMARIS image processing software. Appropriate Gaussian noise removal filter was used depending upon the noise level. The lower threshold level in the histogram was set appropriately to exclude all possible background voxel values. Sum of all the voxel intensities above this threshold level was determined and was considered as the DNA content. We systematically then compared 3D image volume of cells generated using similar imaging conditions. In melanoma tissue sections, we found the cells both immunolabeled for macrophage markers and melanoma markers, as well as the cells immunolabeled either macrophage or melanoma markers. We used Cell Module within IMARIS to identify cells that immunolabeled for both macrophage markers and melanoma markers. The rest of the quantitation procedures were the same as described above.

Deconvolution of 3D confocal image datasets was performed using Huygens software (SVI, Netherlands). For the purpose of deconvolution, the point spread function (PSF) of the optical system was measured for all the emissions using the sub-resolution Fluorescent Microspheres Size Kit (Life Technologies, USA) with identical instrumentation settings. Briefly, a series of confocal optical sections from 0.17 mm beads satisfying the Nyquist sampling criteria was sampled for each emission channel sequentially. The 3D images of these sub-resolution beads were then restored and the PSFs were measured using the Huygens Software. Compiled 3D confocal image data sets were deconvolved with these experimentally measured point spread functions using the iterative deconvolution method which is based on the Classical Maximum Likely Hood Algorithm (CMLE).

### Preparation of MTF cultures for TEM

Cells were grown in culture as described, and then transferred to coverslips (Thermanox coverslips, 15 mm D, Cat#72275–01) and grown for 3 additional days. Cells were then washed with ice cold 0.1 M sodium cacodylate (NaCAC) buffer, pH 7.3 three times for 5 min. Cells were fixed for 1 h at 4 C in 0.5% glutaraldehyde and 4% paraformaldehyde buffered with 0.1 M NaCAC buffer. They were again rinsed 3X with 0.1 M NaCAC at 4 C, and then post-fixed in 1% osmium tetroxide/1.5% potassium ferrocyanide overnight at 4 C in a wrapped container. Preparations were then rinsed with buffer, dehydrated in a graded series of ethanol, and embedded in Embed 812 (Electron Microscopy Sciences). A diamond knife mounted in a Porter-Blum MT-2B ultramicrotome was used to cut 70–90 nm thin sections. Sections were mounted on 200-mesh copper grids and stained with 2% aqueous uranyl acetate + lead citrate. Sections were examined in a Joel Jem 1400 TEM. An Orius SC1000 bottom-mounted CCD camera was used to capture the images. ***s***

### Live Cell Microscopy, Image Acquisition and Quantitation

MTF cultures were grown in glass-bottom microwell dishes (MatTek, #P35G-1.5-14-C) to ~ 50% confluence, then stained with NucBlue live cell stain (ReadyProbes, #R37605) according to manufacturer’s instructions. The experimental protocol used basically followed that of Ohlund et al. [30]. Cells were plated into glass-bottom wells which were either uncoated, or which had been pre-coated with type I collagen. For the live cell migration measurements under various conditions, DeltaVision Elite was utilized along with a heated live cell stage equipped with humidified CO_2_ perfusion system (GE, USA). The live cell data mode integrated into the image acquisition software (Leica Confocal Software LAF) was used for the interactive imaging of live cells. The 3D live cell images were acquired using an Olympus 60X/ 1.4 NA high numerical aperture apochromat oil immersion objective. A sensitive cooled CCD camera was used for capturing images and the images (16 bit) were acquired every 30 seconds for 10 minutes with the appropriate pixel dimensions selected to satisfy the Nyquist sampling criteria. The time series images were compiled and and cell tracking and cell motility measurements were performed using a VOLOCITY (Perkin Elmer, USA) workstation.

## Results

### Isolation and Culture of apparent MTFs

Peripheral blood samples were obtained from patients with cutaneous melanomas under approved IRB protocols with informed consent. OncoQuick porous membrane gradient devices were used to obtain an “enriched CTC” fraction (eCTC; enrichment was ~4-500X vs. PBMCs) as described [26]). In some cases, Ficoll-Paque PLUS gradients were used. The eCTC fractions were then plated onto standard tissue culture dishes and medium containing 10% serum was added. The cultures were rinsed carefully after 24 h (to remove non-adherent cells). At 24 h, many of the adherent cells generally appeared large and “epithelioid” as was reported previously (where we also captured CTCs on filters [26]), and they retained the same basic morphology throughout culturing. Cells were cultured for 3–4 weeks, with medium changed every other day. In most instances, growth was initially quite slow. In initial experiments, cultures consisted of a few thousand cells. The cells seemed to grow better when plated in smaller (25 cm) culture dishes, which eventually provided ~ 5 x 10^5^ cells at ~ 4 weeks. No cells grew in cultures of peripheral blood obtained from normal volunteers.

Upon initial isolation, the populations consisted of ~ 50% “typical” CTCs (i.e., staining for epithelial markers, and not staining for mesenchymal or macrophage markers, or CD45) and 50% apparent MTFs (dual-staining for epithelial and macrophage markers; see below [26]). After 1–2 weeks in culture, the typical CTCs were lost and the populations uniformly were dual-staining. Morphologically, these cultured presumptive MTFs appeared large and spread out, often with diameters ≥ 50 μm. They were flattened (~ 10 μm high), and generally showed abundant pseudopod extensions.

### Immunophenotypic Characterization of Cultured apparent MTFs

**Fig 1:**
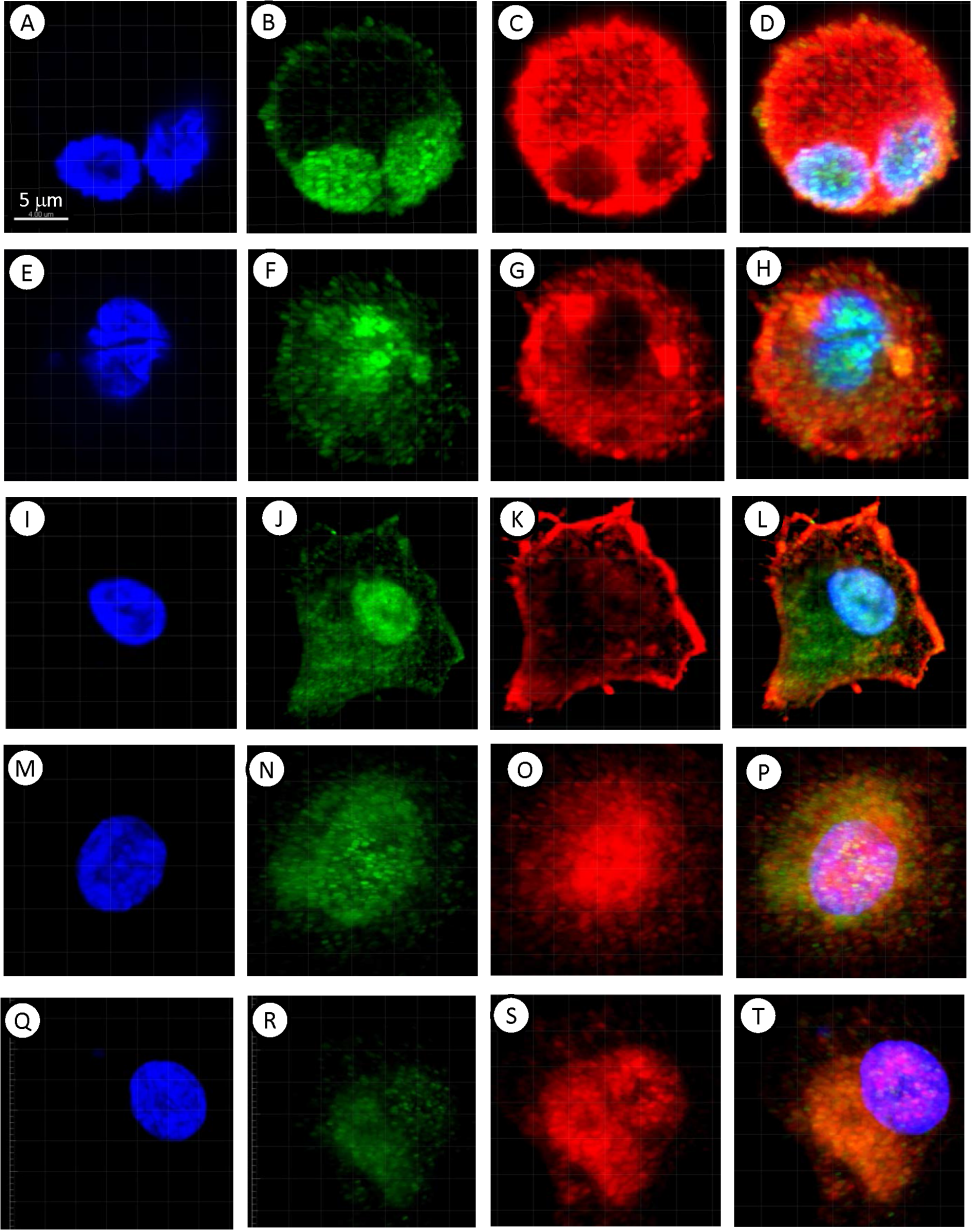
Immunostaining of Cultured MTFs. Representative confocal images of cultured MTFs. Nuclei were stained with DAPI (Blue); individual cells are shown in Panels [**A**,**E**,**I**,**M**, and **Q**]). The same cells were also stained with pairs of various fluorescent markers specific for melanocyte, macrophage, or epithelial differentiation (shown in each row). Panel **[B,C]**: Melanocytic marker ALCAM (Green) and M2- polarization macrophage marker CD204 (Red). Panel **[F,G]**: Pan-Cytokeratin (Green) and M2- polarization macrophage marker CD204 (Red). Panel **[J,K]**: M2- polarization Macrophage marker CD206 (Green) and epithelial cell adhesion molecule EpCAM (Red). Panel **[N,O]**: Melanocyte marker MLANA (Green) and M2- polarization macrophage marker CD204 (Red). Panel **[R,S]**: Melanocyte marker MLANA (Green) and M2- polarization macrophage marker CD206 (Red). Composite images are shown in Panels [**D**, **H**, **L**, **P**, and **T**].

Cultured MTFs were permeabilized and stained with various fluorescent reagents. They stained for standard epithelial markers (**Fig 1**) including pan-cytokeratins (pan-KRTs) and EpCAM (even though melanomas are not derived from epithelium, they routinely express epithelial markers). Interestingly, staining for the pan-KRT marker often showed a preferential nuclear localization (**Fig 1F**: this was also true for the various cell lines examined, and for some MTFs in vivo; see Discussion), although EpCAM staining *showed the standard staining pattern*.

MTFs stained strongly for markers specific for melanocyte lineage, including MLANA, and for cell surface markers often found in melanomas, such as ALCAM (**Fig 1**).

**Fig 2:**
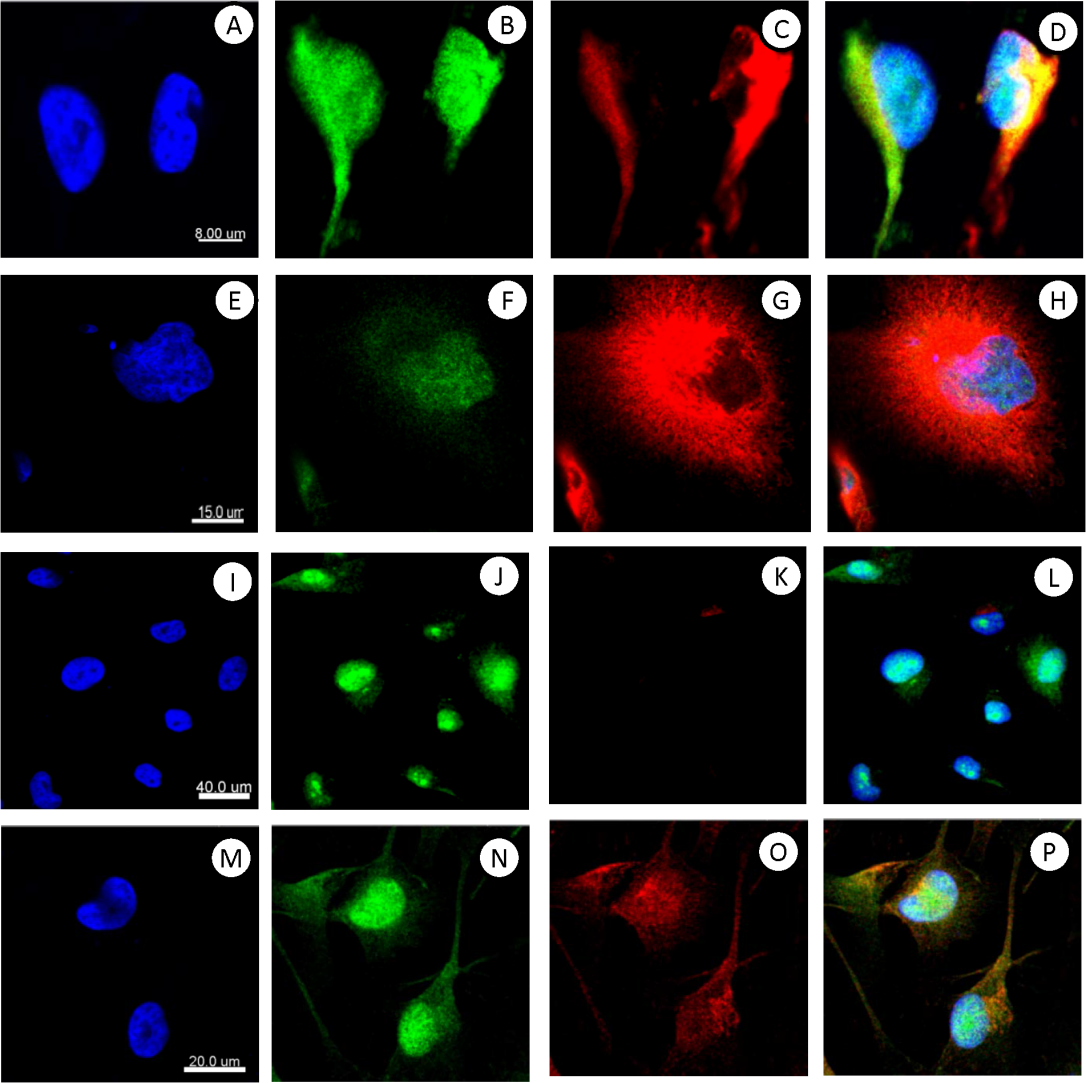
Immunostaining of human melanoma cell lines SK-MEL-28 and SK-MEL-31. Representative confocal images of human melanoma cell lines SK- MEL-28 and SK-MEL-31 are shown. Nuclei were stained with DAPI (Blue) shown in Panels **[A, E, I, and M]**. The same cells were also stained with various fluorescent markers specific for melanocyte, macrophage, or epithelial differentiation. Panel **[B,C]**: SK-MEL-28 stained with M2- polarization macrophage marker CD206 (Green) and melanocyte marker Melan-A. Panel **[F,G]**: SK-MEL-31 stained with M2-polarization macrophage marker CD206 (Green) and melanocyte marker Melan-A. Panel **[J,K]**: SK-MEL-31 stained with pro-carcinogenic cytokine MIF (Green) and pan-macrophage marker CD68 (Red, no signal on the majority of cells). Panel **[N,O]**: SK-MEL-31 stained with M2- polarization macrophage marker CD204 (Green) and epithelial cell adhesion molecule EpCAM (Red). Composite images are shown in Panels **[D,H,L and P]**.

**Fig 3:**
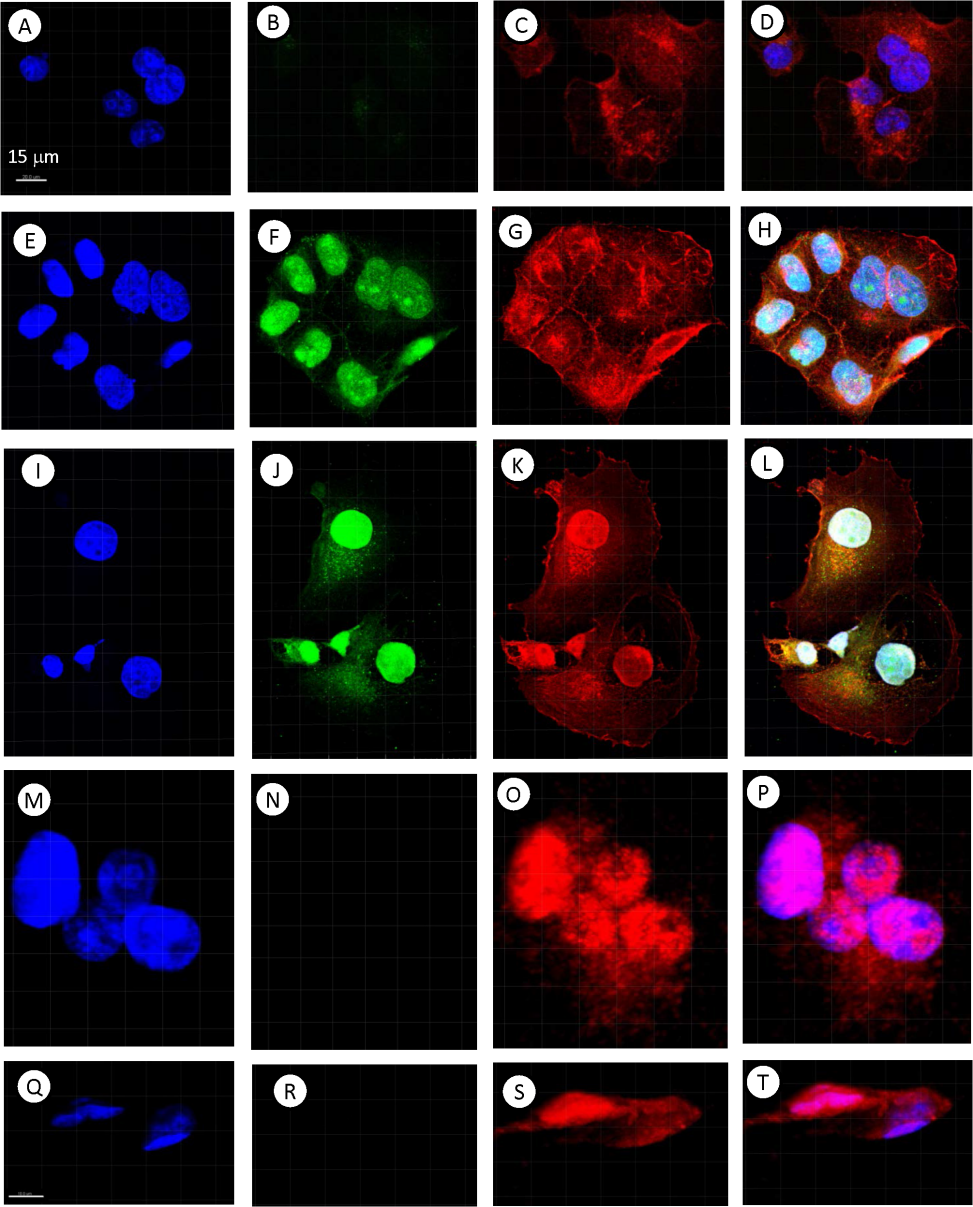
Comparative Immunostaining of human Pancreatic Carcinoma (Panc-1) Cells. Representative confocal images of Human Pancreatic Carcinoma (PANC-1) cells are shown in rows. Nuclei were stained with DAPI (Blue) are shown in Panels [**A**, **E**, **I**, **M**, and **Q**]. The same cells were also stained with various fluorescent markers specific for melanocyte, macrophage, or epithelial differentiation. Panel **[B,C]**: pan-Macrophage marker CD68 (Green, No signal) and M2-polarization macrophage marker CD204 (Red). Panel **[F,G]**: Pan-Cytokeratin (Green) and M2- polarization macrophage marker CD204 (Red). Panel **[J,K]**: M2- polarization macrophage marker CD206 (Green) and epithelial cell adhesion molecule EpCAM (Red). Panel **[N,O]**: Melanocyte marker MLANA (Green, No Signal) and M2- polarization macrophage marker CD204 (Red). Panel **[R,S]**: Melanocyte marker MLANA (Green, No Signal) and M2- polarization macrophage marker CD206 (Red). Composite images are shown in Panels [**D**, **H**, **L**, **P**, and **T**].

The cultured MTFs also routinely stained positive for pan-macrophage markers, including CD14 and CD68. They also stained for a variety of markers indicative of M2 polarized macrophages, including CD204, CD206, and CD163 (**Fig 1**). Most of the staining for CD206 was consistently nuclear (e.g. **Fig 1J**). The human pancreatic ductal epithelial cell line HPDE was used as a normal diploid control for DNA analyses; surprisingly, we found that these “normal” HPDE cells also stained for the M2-polarization markers CD163, CD204 and CD206, although they did not express the pan-macrophage markers CD68 or CD14 (**S1 Fig**). Given this surprising finding, we then examined 3 human melanoma cell lines, as well as the human pancreatic ductal adenocarcinoma (PDAC) cell line Panc-1. The SK-Mel-24, SK-Mel-28, and SK-Mel-31 cell lines showed uniform staining for melanocyte, M2 macrophage, and epithelial markers, with staining patterns analogous to those seen with cultured MTFs. For example, CD206 staining was nuclear, as was seen with cultured MTFs, and pan-KRT staining was largely nuclear (**Fig 2**). Panc-1 cells did not stain for melanocyte markers, but they strongly expressed the epithelial markers pan-KRT (which again was largely nuclear (**Fig 3F**), similar to what was observed in cultured MTFs and SK-Mel cells) and EpCAM. Panc-1 cells also expressed the M2 macrophage markers CD204, CD206, and CD163. As with cultured MTFs and SK-MEL cells, most of the CD206 immunostaining was nuclear in Panc-1 cells (e.g., **Fig 3J**). Like the HPDE cells, Panc-1 cells were also negative for the pan-macrophage marker CD68 (**Fig 3**; see Discussion).

**Fig 4:**
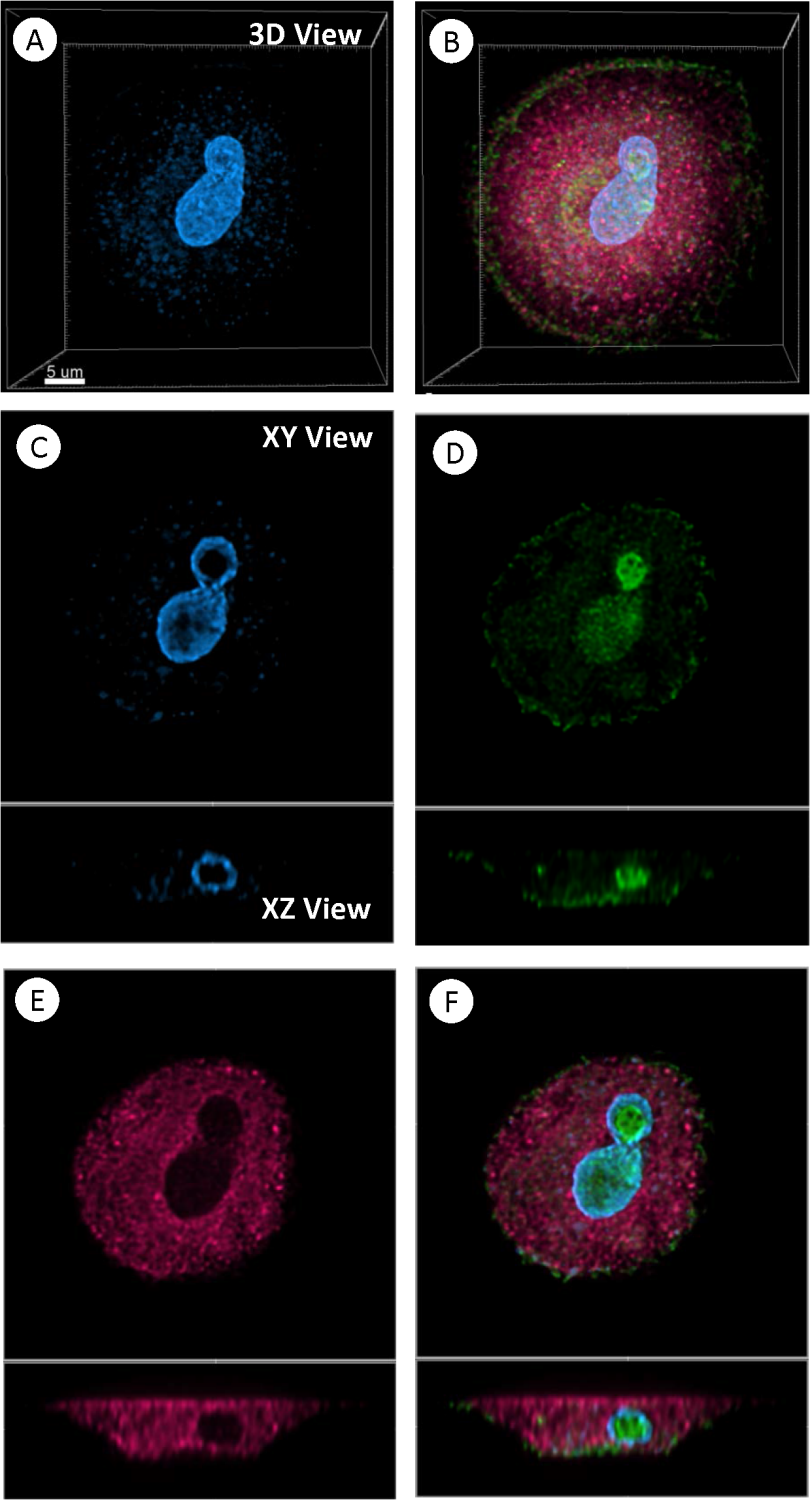
MIF Expression in Cultured MTFs. Shown are representative 3D deconvolved confocal images of Cultured MTFs. Nuclei were stained for nuclei with DAPI (Blue), and immunostained for the pro-carcinogenic cytokine MIF (Green) and the pan-macrophage marker CD68 (Red). Nuclei appeared to have “holes” or “tunnels” through them, and these holes/tunnels stained strongly for MIF. Panels **[A,B]**: 3D projections of DAPI and composite immunostaining. Panels **[C-F]**: Sectional (XY views) views, with XZ views shown beneath them, localizing the accumulated MIF to the holes visible in the nuclei.

**Fig 5:**
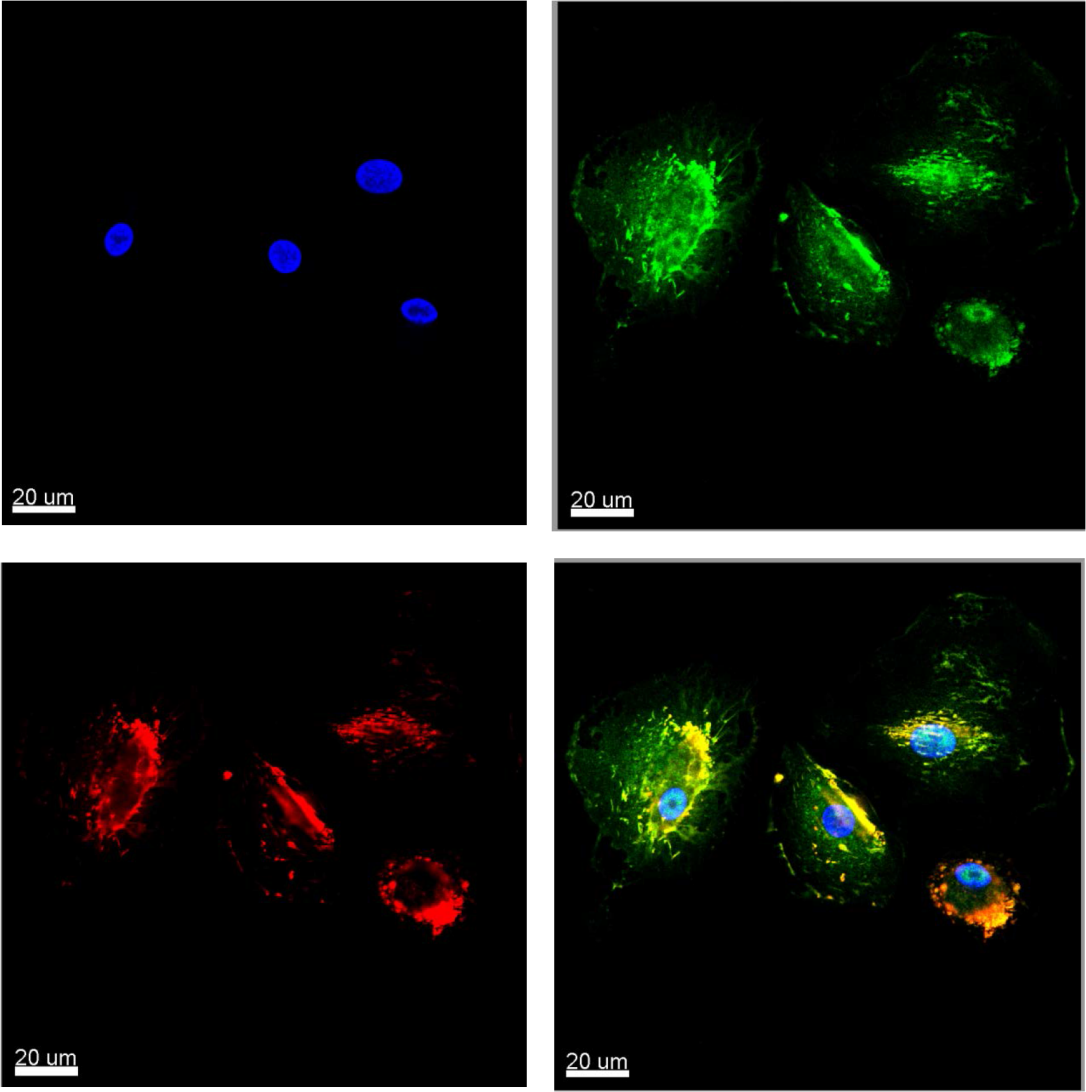
CXCR4 and CD44 Expression in Cultured MTFs. Representative images are shown. Nuclei were stained with DAPI (upper left), and cells were immunostained for CXCR4 (green, upper right) and CD44 (red, lower left). Composite images are shown in the lower right panel.

We examined cultured MTFs for expression of the pro-carcinogenic cytokine MIF, because of MIF’s prominent roles in M2 polarization of macrophages, the tumor microenvironment (TME), and cancer progression. The cultured MTFs routinely stained positively for the MIF (**Fig 4**), with some very intriguing patterns of staining noted. Many of the individual nuclei appeared to have “tunnels” through them; these tunnels (invaginations) were lined by an intact nuclear envelope, and often contained apparently normal cytoplasmic organelles such as ER, golgi, mitochondria, etc. The interior (cytoplasm) within these tunnels stained strongly for MIF, as was determined using 3D reconstructions and deconvolution of confocal images (**Fig 4**; see Discussion). Such tunnels were also observed within melanomas in situ (see below), and they were evident in HPDE cells (although we did not stain HPDE cells for MIF). Given the robust immunostaining for MIF, we also examined the functionally related stem cell markers CXCR4 and CD44. CXCR4 is a non-cognate receptor for MIF [31, 32] and CD44 represents the signaling component of the MIF:CD74 receptor complex [33]. As with MIF, we observed strong expression of CXCR4 and CD44, indicative of pro-carcinogenic activities of these stem cell markers/pathways (Fig 5; see Discussion).

### MTF Ultrastructural Features

**Fig 6:**
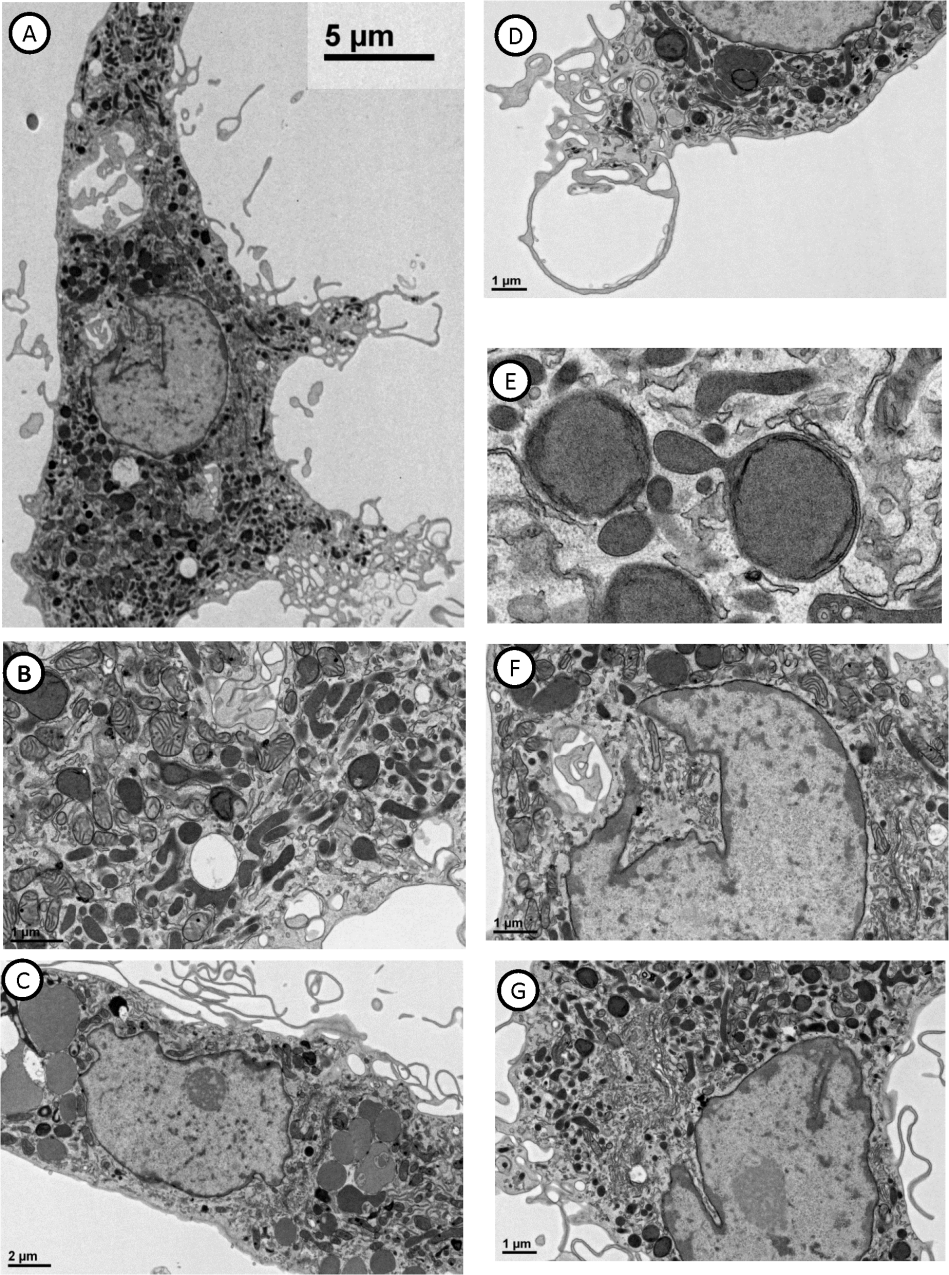
Transmission Electron Microscopy of Cultured MTFs. Cultured MTFs were transferred to coverslips, and grown for ~ 3 days, and then processed and stained as described. Panels **[A-F]** show representative photomicrographs. Essentially all cells appear large (50 μm diameter or larger), and show extensive pseudopod formation. Mitochondria are prominent, as are lysosomes, indicative of active phagocytosis. Heterogeneously-sized autophagic vacuoles and autolysomes at various stages of maturation are readily apparent in most all cells (Panel **E** shows a higher power view of autophagosomes containing micronuclei and chromatin, as well as melanosomes). Cross-sections of “tunnels” through nuclei are evident in many cells (Panel **A**, and Panel **F** shows a higher power view), where subcellular organelles and membranous structures can be seen within the cytoplasmic confines of a tunnel. Melanosomes are prominent in many cells (Panels **B**, **G**, and **F**, for examples).

Cultured MTFs were also examined ultrastructurally using transmission electron microscopy (TEM; **Fig 6**). Essentially all cultured MTFs showed features characteristic of macrophages. The MTFs were generally elongated, large cells (50 μm diameter or larger), which characteristically showed exuberant pseudopod extensions, lamellipodia and exocytosis. They contained large numbers of mitochondria, lysosomes, autophagic vacuoles, and myriad stages of autolysomal breakdown products, including laminated bodies structurally comparable to lysosomes, and various structural remnants (**Fig 6**). A prominent component in essentially all cells were heterogeneously-sized autophagic vacuoles containing chromatin, and often micronuclei. In addition, most MTFs also prominently contained apparent melanosomes and/or premelanosomes ([34]; **Fig 6**). All had about the same density (**Fig 6B, 6G, and 6F**); longitudinally sectioned, they often showed tubular membranous elements containing substructures analogous to those in mature melanosomes. In many MTFs, cross-sections through prominent nuclear “tunnels” were evident (**Fig 6A and 6F**): These tunnels (or invaginations) contained many organelles and membranous structures in their cytoplasmic component.

### MTFs contain DNA derived from Melanomas

Given the prevalence of somatic activating mutations in cutaneous melanomas [35], we first assessed BRAF mutational status in the initial eCTC populations from 11 patients: V600 activating mutations in BRAF were determined using primer-specific RT/PCR and size analysis. 8 of the 11 eCTC populations contained activating BRAF mutations: Six patient samples contained the V600E mutation, 2 patient eCTC samples contained both V600E + V600R mutations, and one additional sample contained the V600K mutation. The cultured MTF populations were also interrogated for the characteristic activating mutations in BRAF. Of 8 MTF cultured populations examined, 5 contained activating BRAF mutations (all 5 had the V600K mutation, 2 samples contained V600K and V600E mutations, and 1 also contained the V600E2 mutation (this percentage reflects the prevalence of BRAF activating mutations in cutaneous melanoma patients). This establishes that these apparent MTFs contain melanoma-derived DNA.

### DNA Content Analyses

After immunophenotypic examination, DNA ploidy was determined using confocal microscopy as previously described [29, 36]. Our basic premise was that if these cultured MTFs may have arisen by fusion, they should exhibit altered ploidy. SK-MEL-24, -28, and -31 melanoma cell lines, as well as Panc-1 and HPDE cells, were also examined, with HPDE cells serving as a standard diploid control.

**Fig 7:**
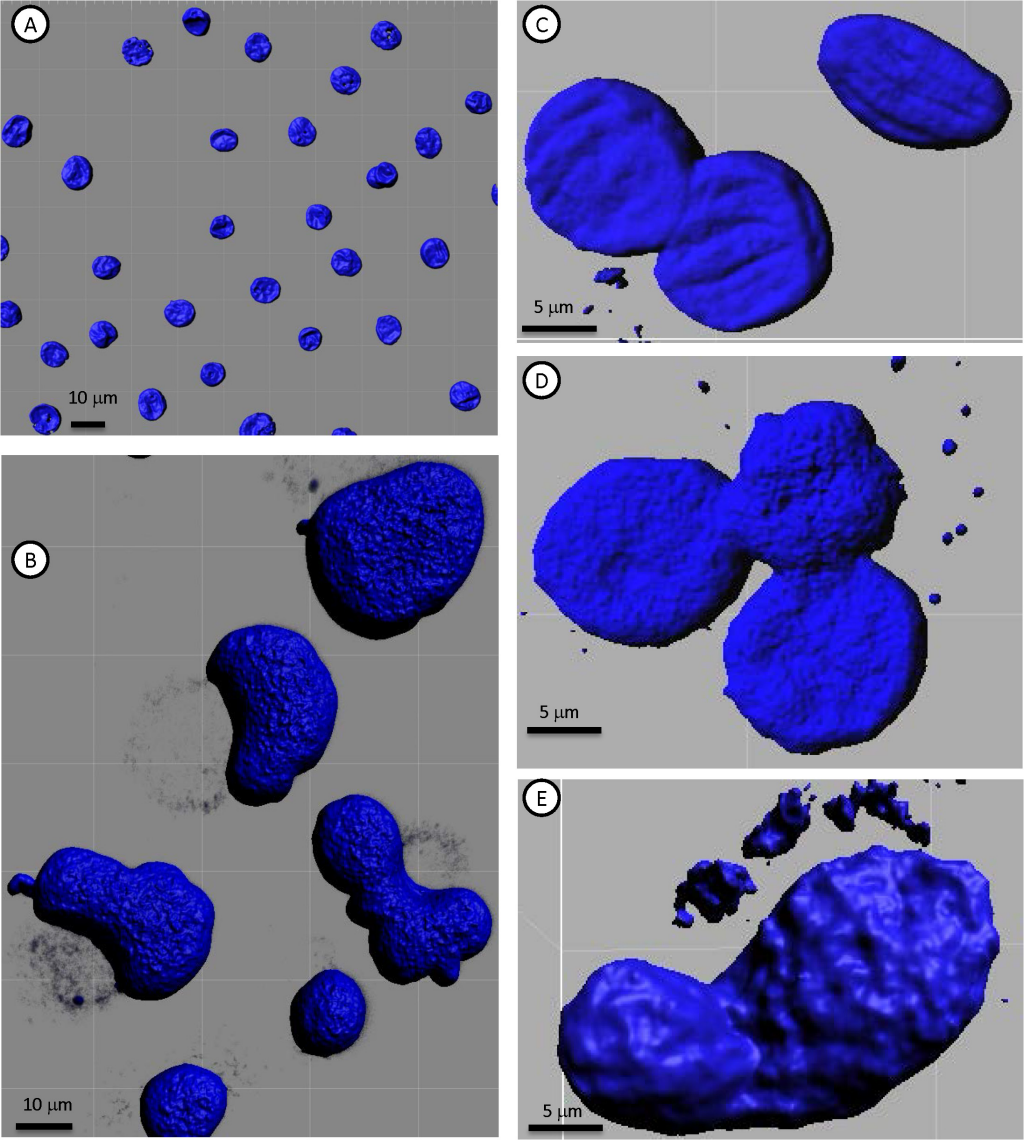
3D Confocal Rendering of DNA in Cultured MTFs. Representative 3D surface rendered confocal images of normal diploid HPDE Cells [**A**], Panc-1 cells [**B**], and cultured MTFs [**C-E**] stained for nuclei with DAPI (Blue). DNA ploidy measurements were performed as described in Methods. HPDE cells serve as a standard diploid control. The cultured MTF populations were very heterogeneous with respect to DNA content (similar results were observed with DAPI and TO-PRO-3 staining). Occasional large cells were bi-nucleate or tri-nucleate, containing 2 or 3 physically attached para-diploid nuclei, and there was also considerable heterogeneity in the size of individual nuclei.

In contrast to their relatively homogeneous immunostaining, the cultured MTF populations were very heterogeneous with respect to DNA content (**Fig 7**; similar results were observed with DAPI and TO-PRO-3 staining). Occasional large cells were binucleate, containing 2 diploid nuclei, and there was considerable heterogeneity in the size and shape of individual nuclei (**Fig 7**).

**Fig 8:**
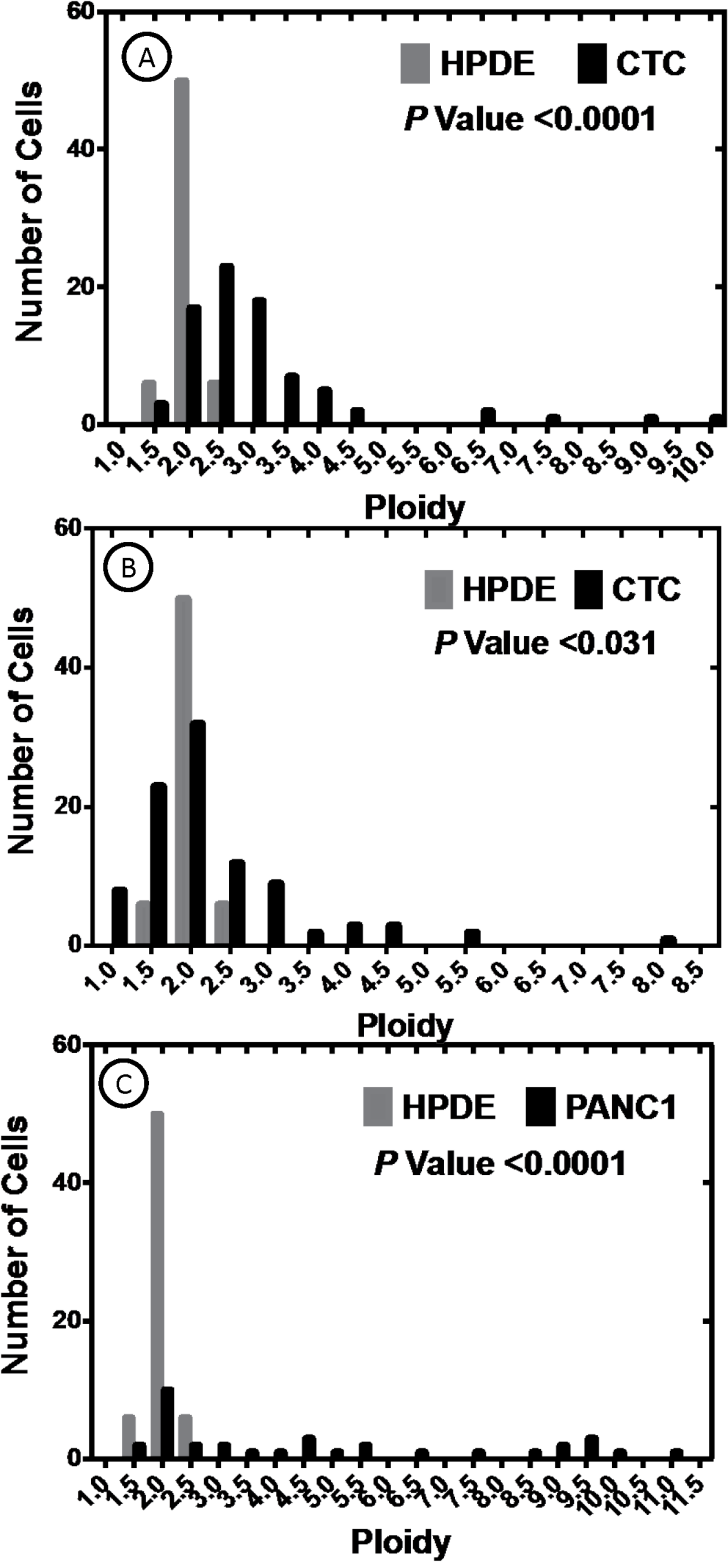
DNA Ploidy Distributions of Cultured MTFs. Graphs **[A-B]**: Populations of Cultured MTFs from 2 representative patients, showed cells with DNA distribution peaks corresponding to “para-diploid” and “para-tetraploid”, but with many aneuploidy cells distributed throughout the range, including some with DNA contents ranging up to 8n or 10n. Also shown on Graphs A-B are control ploidy measurements for normal diploid human HPDE cells. P values show the probabilities that the cultured MTF populations are diploid. Graph [**C**]: Human Panc-1 cells also showed very heterogeneous DNA contents. The distribution of DNA content within the Panc-1 cell populations resembled the DNA distribution observed in the CTC populations.

The populations of cultured MTFs (from 3 separate patients) showed cells with DNA distribution peaks corresponding to ~ diploid and ~ tetraploid, but with many aneuploid cells distributed throughout the range, and some with DNA contents ranging up ~ octaploid or even higher (**Fig 8**).

**Fig 9:**
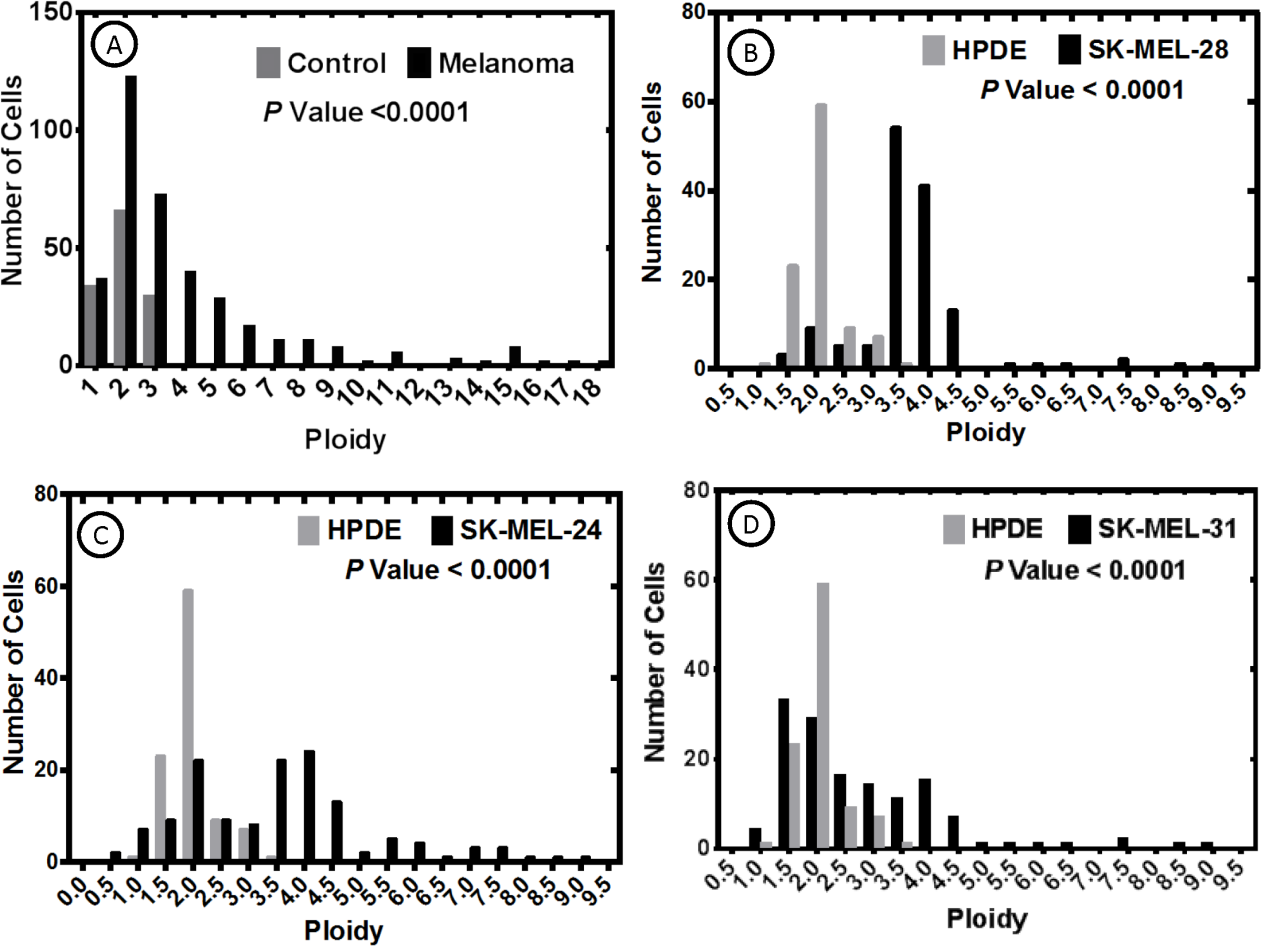
DNA Ploidy Distributions of Melanomas in situ and SK-MEL Cell Lines. DNA content analysis of dual-staining MTFs in primary melanomas in situ was assessed as described, and DNA content distributions were also assessed for the various SK-MEL human melanoma cell lines. DNA contents of all samples differed significantly from that of the normal diploid HDPE cell line. The DNA content of the SK-MEL-24 cells (derived from a metastatic lesion) most closely resembled that observed in melanomas in situ.

Surprisingly, SK-MEL-24, -28, and -31 cells showed similar DNA content distributions, with peaks at 4X and most cells showing > diploid contents (**Fig 9**); occasional cells with ~8X DNA content were also observed. Panc-1 cells also showed very heterogeneous nuclear size and DNA contents, which resembled that observed in the MTF populations (**Fig 8**). HPDE cells showed the expected diploid DNA content (**Figs 8 and 9**).

**Fig 10:**
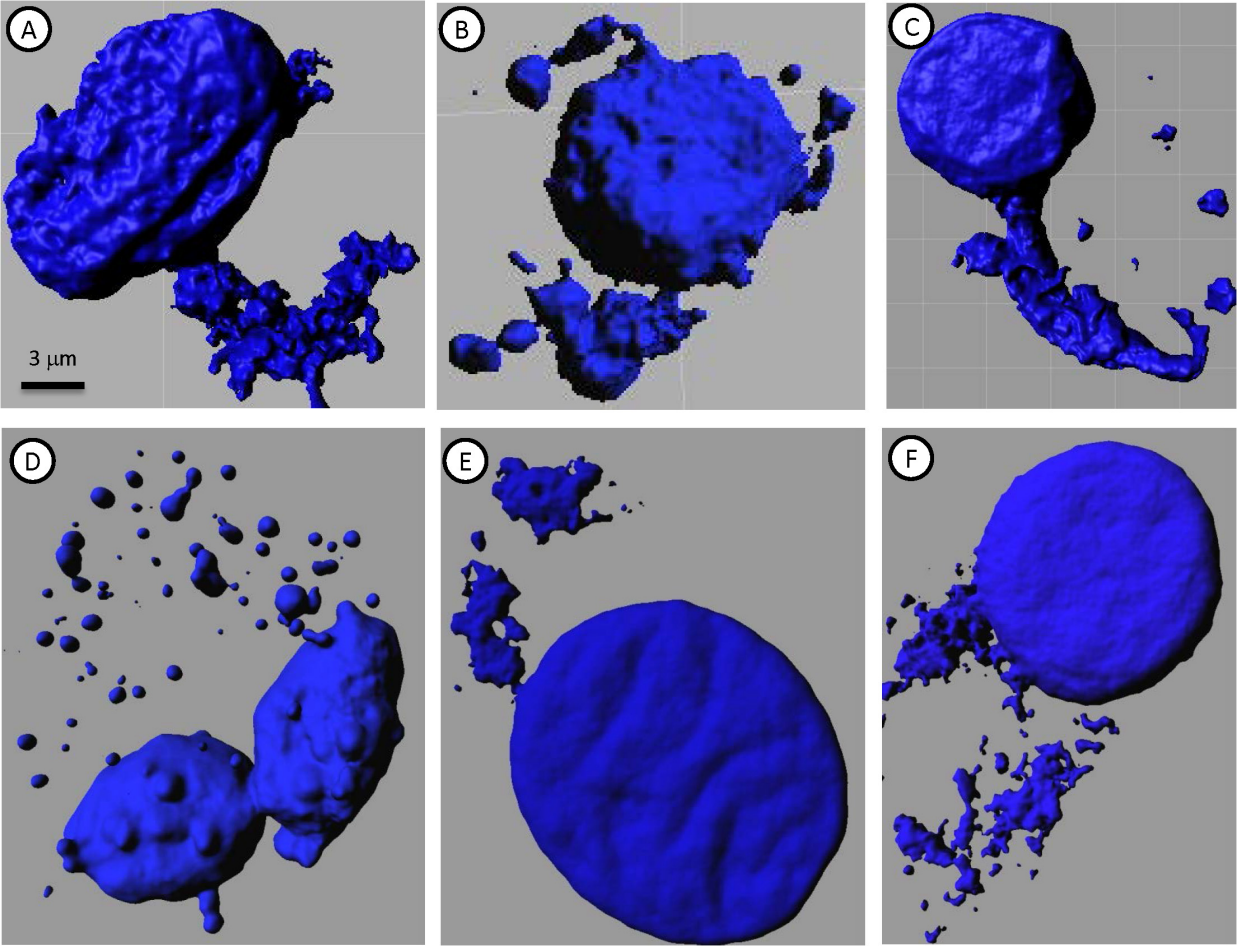
Extrusion of Chromatin in Cultured MTFs. Representative 3D surface rendered confocal images of cultured MTFs stained for nuclei with DAPI (Blue), showing extrusion of DNA from nuclei. There was apparent “Shedding” of DNA from the nuclei into the cytoplasm, which was evident in essentially all of the cultured MTFs. In many cases, this appeared as tubes of chromatin being extruded, in other cases as sheets. Panc-1 cells showed a reduced extrusion of DNA, which appeared much finer than that seen in CTCs.

Even more striking was an apparent “shedding” of DNA from the nucleus into the cytoplasm, which was evident in essentially all of the cultured MTFs (**Fig 10**). In many cases, this appeared as “sheets” of chromatin. This poorly understood process of DNA handling or reconciliation has been referred to as “symphiliosis [37] (see Discussion). In many cells, smaller “clumps” of condensed chromatin were observed in the cytoplasm. This cytoplasmic DNA was contained within autophagosomes, often appearing as micronuclei (see **Fig 6**).

**Fig 11:**
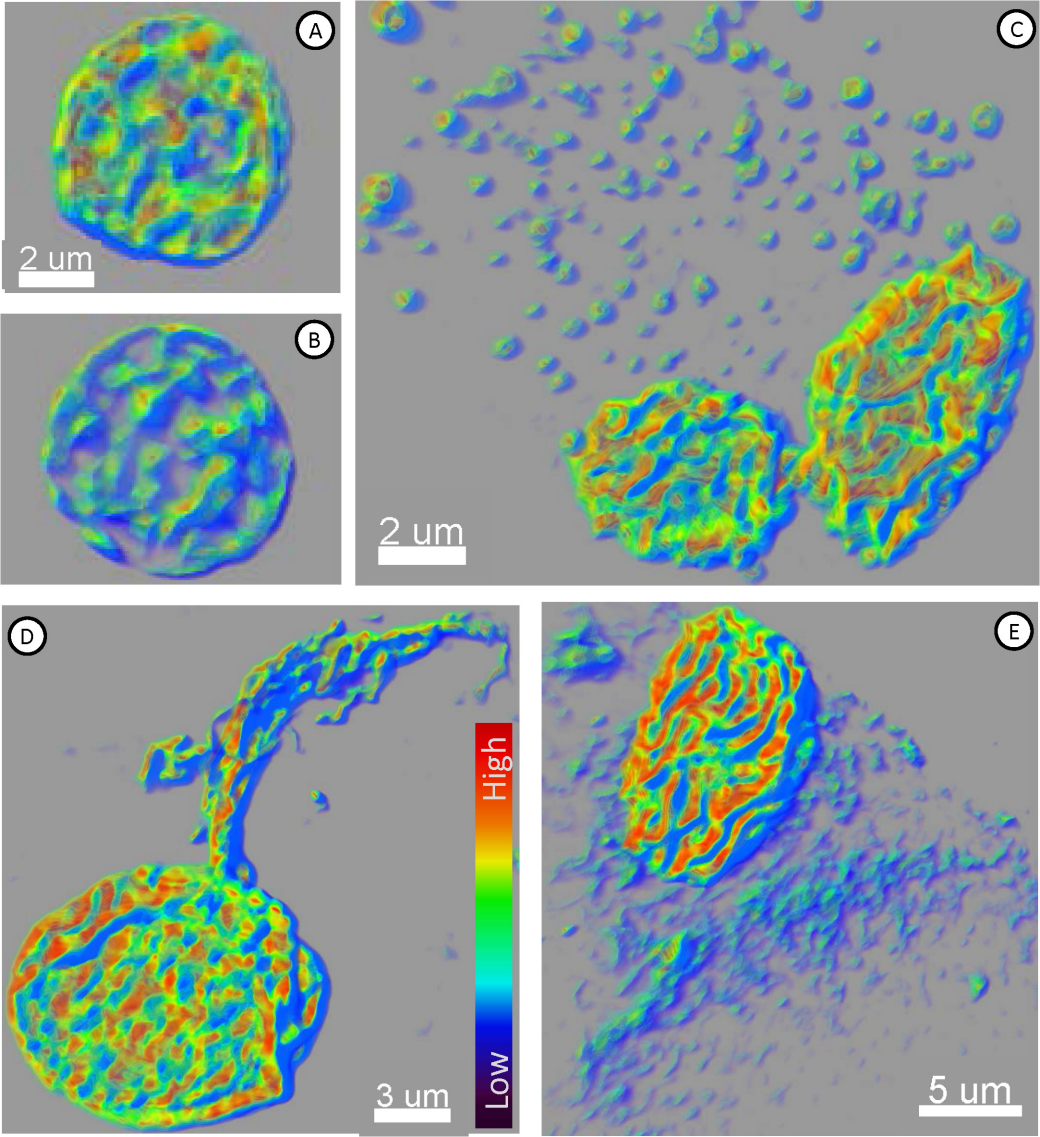
Chromatin Texture Analysis of Cultured MTFs. Representative 3D confocal images of normal human HPDE cells [**A-B**] and MTFs [**C-F**] stained for nuclei with DAPI (Blue). Chromatin texture analysis was performed as described, and the images are color-coded to demonstrate the intensity level (red is most condensed; intensity scale is shown in Panel **D**).

Chromatin texture analysis [29] was then performed, which basically quantifies the DNA signal from the DAPI (or TO-PRO-3) DNA dye. The nuclear chromatin showed obvious signs of organization (**Fig 11**), with many regions of highly condensed (DAPI-intense) chromatin. Such regions have recently been described in fusions between stem cells and somatic cells [22], and have been linked to malignancy in prostate cancer [29]. In most (if not all) cases, the extranuclear chromatin apparently being extruded showed uniform staining indicative of non-condensed chromatin (within autophagosomes).

**Fig 12:**
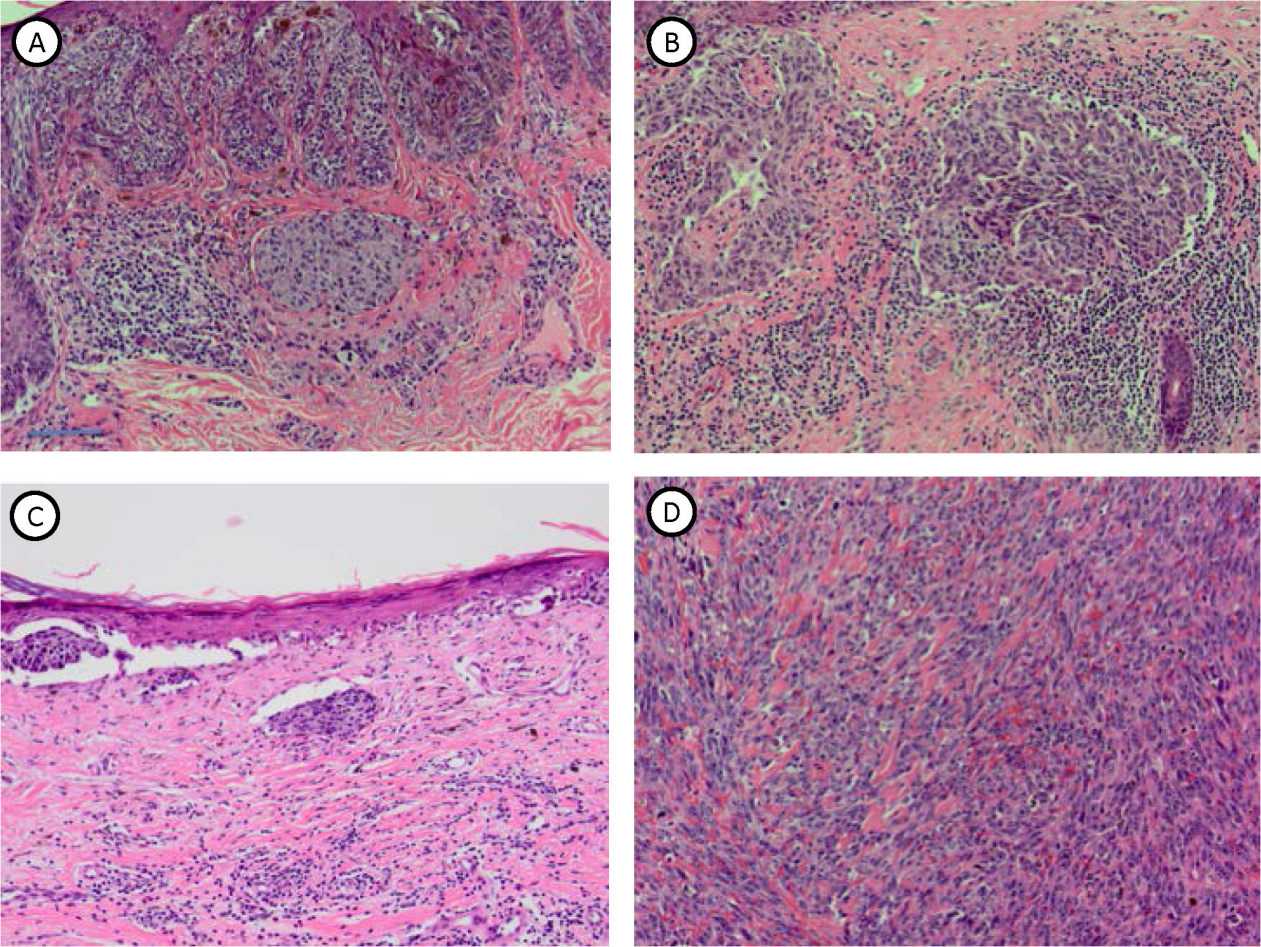
Hematoxylin & Eosin stained tissue sections from cutaneous human melanomas. Formalin-fixed paraffin-embedded tissues from melanoma cases were sectioned and stained with H&E, and examined microscopically. The 4 panels show a range of lesions, including early lesions, which were subsequently examined for “dual-staining” MTFs (macrophage and epithelial markers) using confocal microscopy. Bar in upper left panel represents ~ 100 μm.

**Fig 13:**
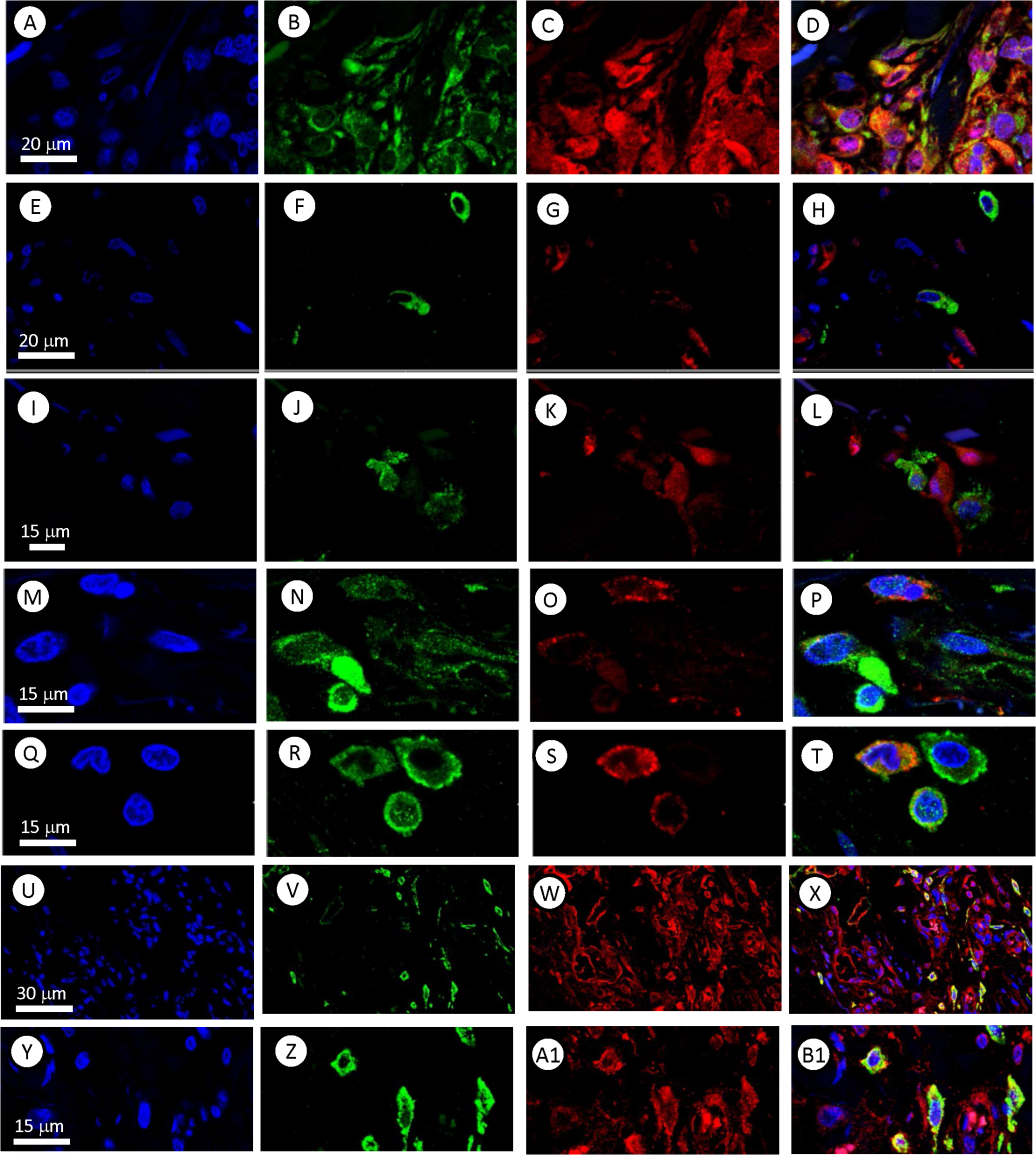
Representative confocal images of MTFs in primary human melanomas. Cells were stained for nuclei with DAPI (Blue), shown in Panels [**A**, **E**, **I**, **M**, **Q**, **U**, and **Y**]. The same cells were also stained with various fluorescent markers specific for melanocyte, macrophage, or epithelial differentiation, and images are shown in rows. Panels **[B,C]**: Melanocyte marker MLANA (Red) and M2- polarization macrophage marker CD204 (Green). Panels **[F,G]**: Melanocyte marker MLANA (Red) and M2- polarization macrophage marker CD206 (Green). Panels **[J,K]**: Melanocyte marker MLANA (Red) and M2- polarization macrophage marker CD163 (Green). Panels **[N,O]**: Melanocytic marker ALCAM (Green) and M2- polarization macrophage marker CD206 (Red). Panels **[R,S]**: M2- polarization Macrophage marker CD206 (Red) and epithelial marker pKRT (Green). Panels **[V,W,Z, and A1]**: M2- polarization Macrophage marker CD163 (Green) and epithelial cell adhesion molecule EpCAM (Red). As is evident, there are distinct populations of cells (in each of 6 primary melanoma specimens examined, as well as the metastatic lesions) which dual-stain for macrophage-melanocyte markers, which are often seen surrounding nests of melanoma cells. These cells also stain for epithelial markers. Composite images are shown in Panels [**D**, **H**, **L**, **P**, **T**, **X**, and **B1**]. Panels underneath Panels [**A-D**] represent XZ views of the panels above.

Since MTFs are present in peripheral blood of melanoma patients, we examined a number of human melanoma FFPE tissue specimens for apparent MTFs (**Fig 12**; 6 primary melanomas and 2 metastatic melanomas were examined). In all primary melanomas, we routinely observed many apparent MTFs, which stained for macrophage (pan- and M2-polarization) markers, as well as for melanocyte-specific (MLANA) and epithelial (EpCAM and pan-KRT) markers (**Fig 13**). In these primary (and metastatic) melanomas, MTFs were clearly identifiable at the periphery of the lesions within nests of melanoma cells. These cells generally appeared irregular and large, with characteristically large nuclei (often multinuclear) and cytoplasmic extensions into the surrounding tissue. In addition to the MTFs, there were also subpopulations of macrophages and melanoma/melanocytes present in the specimens, which did not dual-stain for macrophage/melanocyte markers.

We then assessed the DNA content specifically in the dual-staining apparent MTFs in primary melanomas (**Fig 9**). Their DNA content distribution also showed a very heterogeneous pattern, with many cells in the 4-9n ploidy range, and many additional cells containing much higher DNA contents (up to 18n). It is of interest that the DNA distribution for SK-MEL-24 cells (**Fig 9**), which were derived from a lymph node metastasis, closely resembles that found in apparent MTFs in situ.

**Fig 14:**
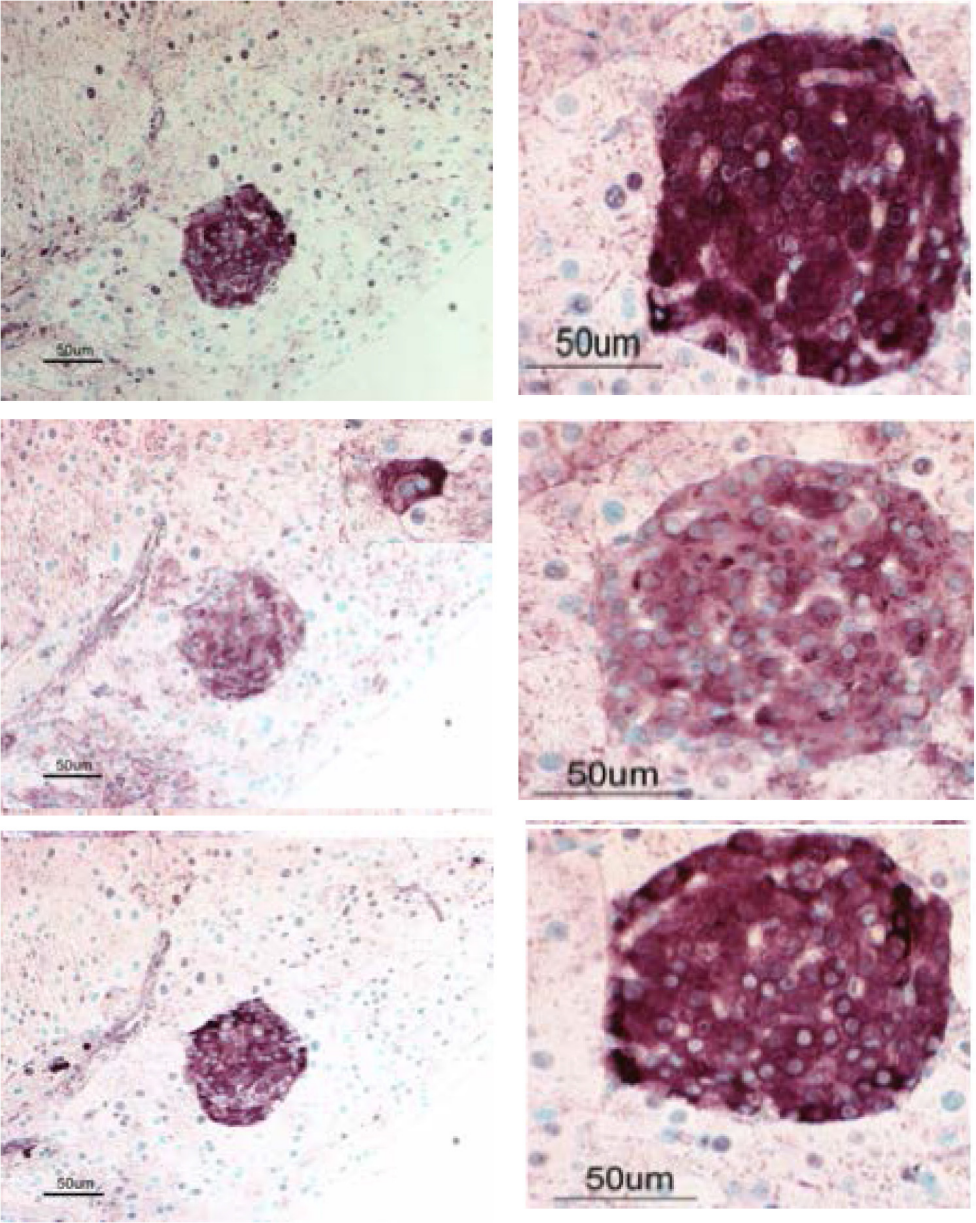
Metastatic Foci after subcutaneous implantation of Cultured MTFs in Athymic Nude Mice. MTFs from 2 separate patient samples were grown in culture for ~ 4 weeks, and 5 x 10^5^ cells were subcutaneously implanted in hind limbs of nude mice. Mice were sacrificed and necropsied 47 days later. Sections were taken from multiple locations and examined for human MTFs. Shown are two distinct metastatic foci in mouse pancreas, stained with antibodies specific for human CD204 (Upper row), MLANA (Middle row), and CD206 (Lower row). Left column shows a low power (20X) view, and Right column shows a higher power (40X) view of 2 distinct foci. Inset in the Left Middle Panel shows a blown-up view of a single binucleate human cell in mouse pancreas, stained for human MLANA. Many of the cells in the foci also contained pigment (melanin), which was visible on standard H&E-stained sections.

An important question was whether the cultured MTFs were capable of metastasis. First, using live cell microscopy, we found that the MTFs were highly motile in vitro (motility coefficient of 0.13 μm^2^/sec). To address metastasis capability, cultured MTFs (5x10^5^) were transplanted subcutaneously in the hind flanks of nude mice, and mice were sacrificed 6–7 weeks later. There was no obvious residual tumor at the injection site. However, there were human MTFs present in subcutaneous tissues in the adjacent skin sections in both mice, and more importantly there were metastatic foci present in the pancreas (**Fig** 14). These foci contained obviously pigmented cells, and were generally characterized by relatively “smooth”, well-defined borders, without obvious aggressive invasion into the surrounding adjacent pancreatic parenchyma (which appeared normal). In addition, there were also often single cells (or nests/aggregates of a few cells) found within stroma in various locations (we see similar features with MTFs from pancreatic adenocarcinoma patients; a manuscript is in preparation). These foci (or cells) dual-stained for human melanocytic markers (MLANA, ALCAM) and human M2-polarized macrophage markers CD206, and CD204 (**Fig** 14), clearly documenting their human origin.

## Discussion

There are a number of findings here which merit discussion: These include; a) the morphologic, ultrastructural, and immunophenotypic (co-expression of epithelial, melanocytic, and macrophage biomarkers) characteristics of the MTF populations, and the presence of melanoma-derived DNA in them; b) potential importance of MIF, and related stem cell markers CXCR4 and CD44 produced by MTFs, in establishing “niches” at distant sites; and c) potential role of ploidy and DNA handling in progression and metastasis.

With regard to the co-expression of markers characteristic of epithelial, melanocytic, and macrophage lineages in the cultured MTFs, all MTFs expressed the standard “epithelial” markers EpCAM and pan-cytokeratin (pan-KRT). The distribution of the expressed EpCAM was that typical of epithelial cells. However, much of the expressed pan-KRT we observed was localized to the nucleus. Although the meaning of this is not clear, distinct molecular forms of KRT have been described in the nucleus, which do not possess a filamentous structure [38]. This largely nuclear localization of KRT was also observed in SK-MEL cell lines as well as Panc-1 cells. Nuclear pan-KRT localization was also occasionally observed in melanomas in situ.

The MTFs expressed a number of markers characteristic of melanocytic differentiation or melanoma. For example, they expressed MLANA (Gene ID#2315, Melan A), which is involved in melanin biogenesis. Ultrastructurally, the MTFs prominently contained apparent melanosomes (and/or premalanosomes), based on previous detailed descriptions [34]. We also note than MLANA mRNA serves as an excellent positive biomarker in peripheral blood from early stage melanoma patients [26], where it could represent circulating MTFs. MLANA expression is also useful for diagnosis of invasive cutaneous melanomas [39]. The MTFs also expressed ALCAM (Gene ID#214), a marker which is characteristically expressed in melanomas [40, 41]. Expression of these markers was consistent across all cells in the populations, and does not seem to reflect promiscuous expression.

Given the prevalence of activating BRAF mutations in cutaneous melanomas [35], we interrogated eCTC and MTF cultures for somatic activating mutations in BRAF. 8 of 11 of the initial eCTC populations contained activating mutations in the kinase domain of BRAF, a somatic mutation characteristic of many primary melanomas. When 8 additional MTF cultures were interrogated after 4 weeks in culture, 5 of them also contained the activating V600E mutations. This establishes that what appear to be macrophages contain DNA derived from melanomas.

The MTFs uniformly expressed a number of macrophage markers, many of which are characteristic of M2-polarized macrophages, such as CD163, CD204, and CD206. CD163 (Gene ID#9332) is a member of the scavenger receptor cysteine-rich superfamily, which may reflect proinflammatory cytokine production, and there are various reports linking its expression with poor prognosis in various cancers [42, 43]. CD204 (Gene ID#4481) is officially known as MSR1, the class A macrophage scavenger receptor type 1. It is a functional receptor which mediates the endocytosis of low density lipoproteins, implying lipid metabolism, and its expression has also been linked with various cancers as well as with intralymphatic metastasis [44] CD206 (Gene ID#4360) is MRC1, the mannose receptor, C type 1; it is involved in glycoprotein metabolism, and curiously has also been shown to be involved with CD44 in lymphatic trafficking [45]. We believe that expression of these receptors may indicate use of alternative energy sources by transformed cells [46–51]. It is also of interest that while Panc-1 and HPDE cells strongly express CD204 and CD206, they do not express pan-macrophage markers like CD68 (Gene ID#968) or CD14 (Gene ID#929): CD68 and CD14 are unlikely to contribute to altered metabolic requirements, although it is of interest that activating BRAF mutations do alter metabolic pathways in melanoma [52].

Given that MTFs were found in peripheral blood of melanoma patients, we also examined human melanoma specimens for apparent MTFs. MTFs were readily identifiable in primary melanomas, with strong staining for melanocyte (MLANA and ALCAM), macrophage (CD204, CD206), and epithelial (pan-KRT, EpCAM) markers. It is of interest that CD206 expression in MTFs in primary melanomas generally did not show the nuclear localization observed in cultured MTFs or cell lines (compare **Fig 13R** with **Fig 1J**). There is a report which described “stealth” melanoma cells in histology negative sentinel lymph nodes [53]; these may well have represented MTFs.

The M2-polarization of cultured MTFs may have significant ramifications. M2-like macrophages are responsible for collagen degradation through a CD206-mediated pathway [54], and tumor associated macrophages (TAMs) generally acquire an M2-like phenotype that plays important roles in many aspects of tumor growth and progression [55–58]. M2-polarized TAMs have also been found to promote the EMT in various carcinomas [59, 60].

In fact, there are a growing number of reports of expression of macrophage markers on various types of cancer cells; this does not simply reflect “plasticity” in gene expression, but rather a characteristic acquisition of macrophage systems. For example, CD163 expression on rectal cancer cells is associated with early local recurrence and reduced survival time [61]. CD163 expression by breast cancer cells is related to early distant recurrence and reduced survival time [62], and breast cancer cells expressed CD68. In this regard, Shabo & Svanvik [63] reported that 48% of breast cancer cells expressed CD163, and that 31% of rectal cancer cells expressed it. CD163 was again associated with early distant recurrence in breast cancer, and with local recurrence in rectal cancer, and with reduced survival times in both. Expression of DAP12, a macrophage fusion receptor, was also associated with advanced tumor grade and higher rates of skeletal and liver metastasis, and overall shorter distant recurrence-free survival.

### Macrophage Tumor Cell Fusions

Pawelek and co-workers [17, 64–66] have long espoused the concept of MTFs (or more generally myeloid cell-tumor cell fusions) and their potential role in metastasis, and they recently described a CNS melanoma which may have represented a metastatic MTF lesion [18]. Fusion between intestinal epithelial cells and macrophages in a cancer context results in nuclear reprogramming [19]: MTFs reportedly retain a transcriptome identity characteristic of both parental derivatives, while also expressing a unique subset of transcripts. Ding et al. [24] reported that MTFs may even acquire cancer stem cell properties in breast cancer cells. They observed aberrant CD163 expression in breast cancers, and created fusions between M2-polarized macrophages and breast cancer cell lines. The MTFs showed a CD44^+^CD24^-/low^ phenotype, and demonstrated increased migration, invasion, and tumorigenicity (but reduced proliferative ability) and they over-expressed EMT genes. In a related vein, other have described in vivo fusion of human cancer cells with hamster stromal cells, resulting in tumors which express the tumors oncogenic driver mutations as well as stromal characteristics [67]. Macrophage-fusion cells are not exclusive to cancers; fusion between hematopoietic and epithelial cells in adult human intestine has also been described [68].

### MIF in the TME and Cancer Progre*ssion*

Another noteworthy observation was the expression of MIF by MTFs, since MIF plays critical roles with M2 polarized macrophages, the TME, and it has been implicated in diverse pathways involved in cancer progression.

MIF levels are associated with an increased incidence of prostate cancer [69], non small cell lung cancer [31], squamous cell carcinomas of the nasopharynx [70] and esophagus [71], breast cancer [72], colorectal cancer [73], hepatocellular carcinoma [74] and several other cancers [75]. MIF produced in the TME also regulates angiogenesis in a melanoma model [76]. MIF serves as a non-cognate ligand for CXCR2 and CXCR4 [31, 32]: MIF (and CXCR4) in the TME are adverse prognostic indicators in esophageal cancer [71], and it can induce CXCR ligand and regulators of macrophage infiltration like CD44 [77]. Here we found that cultured MTFs also expression CXCR4 and CD44 in addition to MIF (Fig 5). In PDAC, MIF has been shown to induce the EMT [78, 79], enhance tumor aggressiveness, and predict clinical outcome in resected PDACs [80]. M2 polarized TAMs have specifically been shown to have prognostic importance in pancreatic ductal adenocarcinoma (PDAC) [57, 81], and targeting TAMs decreases tumor-initiating cells, relieves immunosuppression, and improves chemotherapeutic responses [82]. MIF controls alternative activation of TAMs to M2-polarization [78]; In turn, co-culturing M2-polarized TAMs with PDAC cells strongly induces the EMT [79].

MIF expression is up-regulated during hypoxia generally associated with the TME, via an HRE found in the 5’-UTR of the gene [83, 84]. Proteomic and tissue array profiling has identified elevated hypoxia-regulated proteins in microdissected PDAC nests vs. normal ducts [85], prominently including MIF, which showed excellent ROC curves in discriminating PDACs. MIF is a direct transcriptional target of HIF1, and loss of MIF results in inefficient HIF1 stabilization induced by hypoxia [86]. Intracellularly, MIF is also stabilized by complexing with HSP90 chaperone [72, 75]. Cancer cells contain constitutive endogenous MIF-HSP90 complexes, and inhibition of HSP90 function results in apoptosis, which can be overridden by ectopic MIF expression In fact, the metastasis-promoting CD44 in PDACs is actually the signaling component of the MIF-CD74 receptor complex [33]. MIF signaling through CD74 promotes sustained ERK activation, which corresponds to the main outcome from Ras mutations, mutations which figure so prominently in PDAC (see [87]). Inhibition of MIF using siRNAs leads to apoptosis in PDAC cells [88]. Long et al. [89] developed a unique mouse model for PDAC lymphatic metastasis. They developed a subline from the BxPC-3 PDAC cell line via serial passages in nude mice. The subline showed increased migration, invasion, and invasive ultrastructural characteristics. Metastasis-related gene alterations found in the subline were quite limited but included up-regulation of MIF.

Here, MIF was characteristically found within “tunnels” in MTF nuclei, both in cultured MTFs (**Fig 4**) as well as in apparent MTFs in primary melanomas (**Fig 13**), and in the SK-MEL cells lines. These “tunnels” often contain intact cytoplasmic organelles, and they are lined by an intact nuclear envelope. We are not aware of any previous reports describing similar nuclear tunnels, or of any focal, peri-nuclear localization of MIF, although an interaction between NME1 and MIF has been reported [90]; NME1 was found to alleviate suppression of p53 activity by MIF, by disrupting binding of p53 by MIF.

Another prominent finding was the heterogeneous polyploidy DNA content found in cultured MTF preparations (**Fig 8**), with a significant proportion of the MTFs containing 4n to 8n (or higher) DNA content.

On the one hand, there is clear evidence linking DNA index to prognosis of several cancers.

Using PDAC as an example, DNA index has been shown to be a strong prognostic factor in PDAC patients [91, 92] where 50–75% of patients showed non-diploid DNA contents. There is also a clear relationship between DNA content and survival in PDAC patients. Lymph node involvement was seen in 36% of patients with diploid tumors, vs. 79% of those with aneuploid tumors. 32% of patients with a diploid tumor survived at least 1 year, whereas none of the aneuploidy patients did [93], and aneuploidy showed a significant association with decreased cumulative survival. Tsavaris et al [94] found that PDAC patients with ploidy score > 3.6 had 5X higher probability of death compared with patients with ploidy score < 2.2, and those with an intermediate ploidy score 2.2–3.6 had a 6.3X higher probability of death compared with patients with ploidy score < 2.2. A similar relationship was found for patients with late stage colorectal cancers [95].

On the other hand, the route by which polyploidy occurs may have a major impact on its consequences, as has recently been hypothesized based on lessons from plants [37]. There, polyploid cells arising as “Allopolyploids” have far different characteristics than those arising as “autopolyploids” [27, 37]. When fusion of 2 different cell types occurs, two distinct cellular programs need to be merged somehow. This process has been referred to symphiliosis, the process of intracellular reconciliation [37]. This suddenly produces new clones with emergent phenotypes. Cancer cells can transduce adjacent TME cells in vivo, and it has been suggested that in vivo fusion discloses genes implicated in tumor progression, as well as gene families coding for the organoid phenotype [67]. Here, we note that the MTFs present a surprisingly uniform immunophenotypic profile, in spite of the often huge differences in DNA content. They appear to be undergoing cellular reconciliation, with apparent shedding of massive sheets (and/or clumps) of cytoplasmic chromatin, with the extranuclear DNA being handled by nucleophagy [96] within autophagosomes which have sequestered chromatin and even micronuclei. Many aspects of micronuclei formation have been detailed [97], and degradation of micronuclei via autophagosomes has previously been reported [98], where it was speculated that removal of micronuclei may contribute to the genome-stabilizing effects of autophagy. Nucleophagy has been reported in various laminopathies [99] and seems to be beneficial for cell survival [99, 100].

Chromatin texture analysis also identified focal areas of condensed “DAPI-intense” chromatin staining. Similar regions have been reported in fusions between embryonic stem cells and somatic cells [22], a setting which is currently being examined in the context of “reprogramming” [20, 21]

There are many examples of naturally occurring polyploidization (e.g., liver and skin; [101, 102]), and polyploidization and cell fusion appear to contribute to wound healing in the adult Drosophila epithelium [103]. However, senescence and autophagy are also thought to be intimately involved in the emergence of self-renewal potential in surviving cells that result from a process termed “depolyploidization” [104]; Erenpreisa and co-workers have suggested that genotoxic resistance is afforded through a programmed life-cycle-like process which intimately unites senescence, polyploidy, and “stemness” (self-renewal) as steps to “immortality” for cancer cells. The process seems to involve macroautophagy-aided elimination of chromatin, which somehow entails sorting out what will be eliminated [105]. While we find a “diploid” peak in DNA content in the MTF populations, it seems more appropriate to term it “paradiploid”, since it is known that polyploid tumor cells elicit paradiploid progeny through depolyploidizing divisions and regulated autophagic degradation [106]. The process appears to involve substantial nuclear volume increases with spatial shifts of chromosome territories in nuclei of radiation-induced polyploidizing tumor cells; this reflected generation of large intra-nuclear chromosome territories and their repositioning prior to genome reduction [107] (an interesting question is whether radiotherapy might influence fusion frequency in patients). Although many aspects of these various processes are not well-defined, it seems clear that here they ultimately produce a population of MTFs which are capable of sustained growth and metastases after implantation into nude mice. As noted with plants (allopolyploids vs. autopolyploids), the way in which polyploidy arose in these apparent MTFs may be an important determinant for how they handle their DNA, so that the typical “depolyploidization” process does not occur.

We suggest that MTFs participate in the earliest stages of the metastatic cascade. We hypothesize that they form very early on at the periphery of melanomas, locally induce the EMT, and readily enter the circulation, subsequently colonizing distant sites and secreting cytokines such as MIF. This produces “niches” (mini-TMEs) suitable for establishment of metastatic lesions by MICs, which may be liberated cancer stem cells.

## Supporting Information

S1 Fig Expression of M-2 Macrophage Markers in normal Human Pancreatic Ductal Epithelial (HPDE) cells. Representative confocal images of normal HPDE cells are shown. Nuclei were stained with DAPI (Blue) and cells with various fluorescent markers specific for macrophage or epithelial differentiation. Panels **[A-B]** show DAPI staining [**A**] and immunostaining (Red) for the M2- polarization macrophage marker CD206 [**B**]. Panels **[C-D]** show DAPI staining [**C**] and immunostaining (Red) for the M2- polarization macrophage marker CD204 [**D**]. Panels **[E-H]** show DAPI staining [**E**], immunostaining (Green) for the M2-Macrophage marker CD163 [**F**] and immunostaining (Red) for the epithelial marker EpCAM [**G**]. Panel [**H**] shows the composite image.
(TIFF)

S1 Table Antibodies used for the Immunohistochemical Staining. (DOCX)
