# Supplementary figures and images for: Macrophage-Tumor Cell Fusions from Peripheral Blood of Melanoma Patients
Source: PLoS One. 2015 Aug 12;10(8):e0134320. doi: 10.1371/journal.pone.0134320 (PMC4534457; doi:10.1371/journal.pone.0134320)

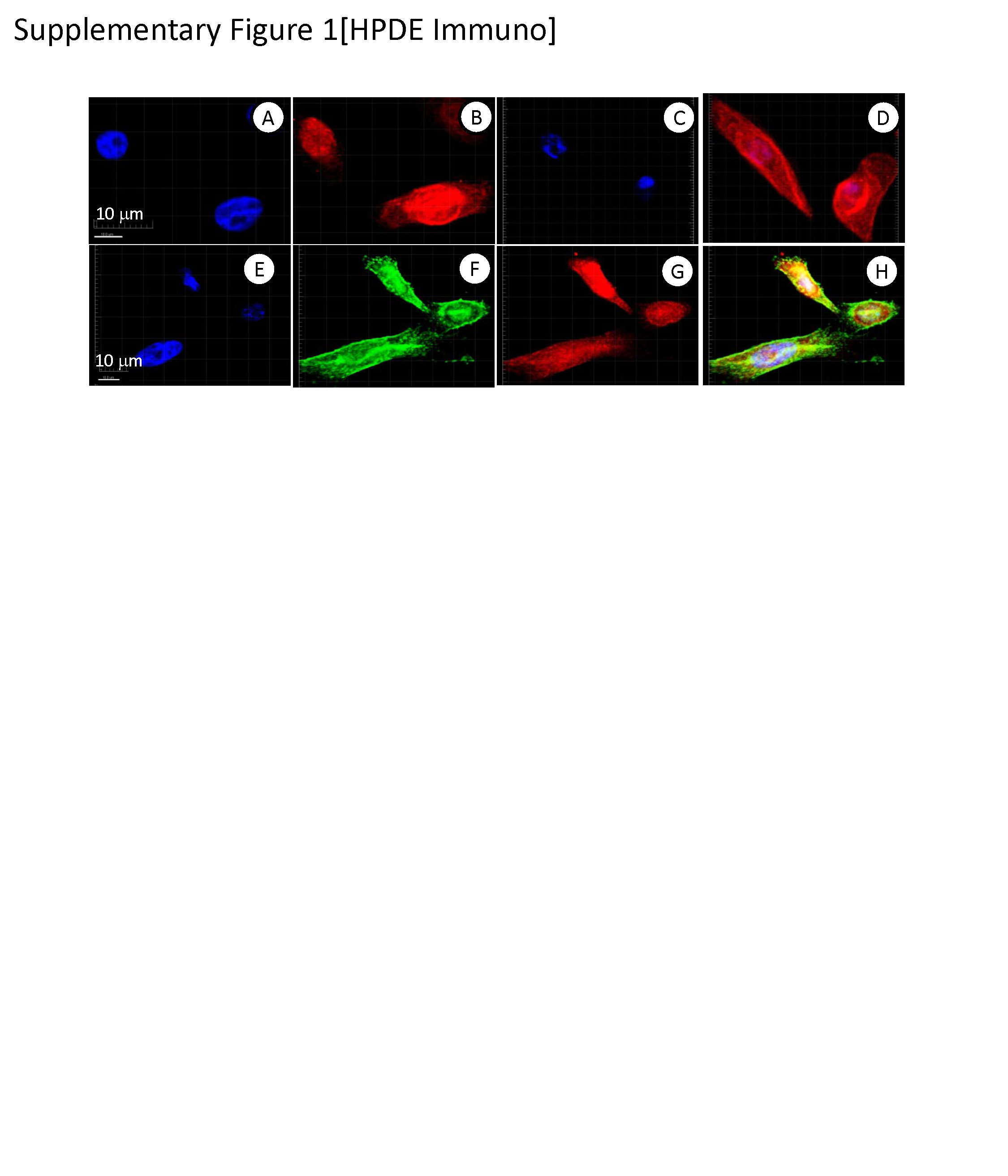

Supplement: S1 Fig — Representative confocal images of normal HPDE cells are shown. Nuclei were stained with DAPI (Blue) and cells with various fluorescent markers specific for macrophage or epithelial differentiation. Panels [A-B] show DAPI staining [A] and immunostaining (Red) for the M2- polarization macrophage marker CD206 [B]. Panels [C-D] show DAPI staining [C] and immunostaining (Red) for the M2- polarization macrophage marker CD204 [D]. Panels [E-H] show DAPI staining [E], immunostaining (Green) for the M2-Macrophage marker CD163 [F] and immunostaining (Red) for the epithelial marker EpCAM [G]. Panel [H] shows the composite image. (TIFF) [file pone.0134320.s001.tiff]
