# Supplementary material for: Macrophage-Tumor Cell Fusions from Peripheral Blood of Melanoma Patients
Source: PLoS One. 2015 Aug 12;10(8):e0134320. doi: 10.1371/journal.pone.0134320 (PMC4534457; doi:10.1371/journal.pone.0134320)
Supplement: S1 Table — (DOCX) [file pone.0134320.s002.docx]

| Supplementary Table 1. Primary Antibodies used for Immunochemical Staining | | | |
| --- | --- | --- | --- |
| Antibody Name | Company | Cat# | Source |
| CD14 | BD | 555398 | mouse |
| CD45 | Santa Cruz | Sc-59071 | rat |
| CD63 | Millipore | CBL553 | mouse |
| CD68 | Bioss | bs-0649R | rabbit |
| CD68 | Abcam | Ab955 | mouse |
| CD81 | Millipore | CBL579 | mouse |
| CD163 | Abcam | Ab87099 | rabbit |
| CD204 | SB | 10427 | mouse |
| CD204 | Bioss | Bs-6763R | rabbit |
| CD206 | Bioss | Bs-2664R | rabbit |
| EpCAM | Millipore | OP-187 | mouse |
| MIA | Santa Cruz | Sc-28868 | rabbit |
| MIF | Santa Cruz | Sc-20121 | rabbit |
| KRT (Pan-Cytokeratin) | Santa Cruz | Sc-15367 | rabbit |
| ALCAM | Santa Cruz | Sc-25624 | rabbit |
| Melan-A | Santa Cruz | Sc-20032 | mouse |
| Melan-A | Novus | NBP1,30151 | rabbit |
| Fluorescently-labeled AffiniPure secondary antibodies from Enco Scientific Services Limited | | | |
| AlexaFluor 488 anti-mouse IgG | | 115-545-062 | goat |
| AlexaFluor 594 anti-mouse IgG | | 115-585-062 | goat |
| AlexaFluor 488 anti-rat IgG | | 112-545-062 | goat |
| AlexaFluor 594 anti-rat IgG | | 112-585-062 | goat |
| AlexaFluor 488ati-rabbit IgG | | 711-545-152 | donkey |
| AlexaFluor 594 anti-rabbit IgG | | 711-585-152 | donkey |
| Fluoresecently-labeled secondary antibodies from Molecular Probes Life Technologies | | | |
| AlexaFluor 488 anti-goat IgG | | A11078 | rabbit |
| AlexaFluor 488 anti-rabbit IgG | | A11034 | goat |
| AlexaFluor 594 anti-mouse IgG | | A21203 | donkey |
